# Isoform-specific roles of QKI-6 and QKI-7 direct Schwann cell lineage progression and enhance peripheral nerve regeneration

**DOI:** 10.1038/s12276-026-01708-0

**Published:** 2026-05-01

**Authors:** Han-Seop Kim, Jae Yun Kim, Ji-Young Lee, Ji Eun Jeong, Binna Seol, Ji Eun Choi, Yee Sook Cho

**Affiliations:** 1 Stem Cell Research Laboratory, Immunotherapy Research Center, Korea Research Institute of Bioscience and Biotechnology (KRIBB), Yuseong-gu, Daejeon, South Korea; 2 Department of Bioscience, KRIBB School, University of Science & Technology, Yuseong-gu, Daejeon, South Korea

**Keywords:** Stem-cell research, Regeneration

## Abstract

Peripheral nerve injury presents a major therapeutic challenge owing to limited endogenous repair and incomplete functional recovery. Schwann cells (SCs), the principal glia of the peripheral nervous system, support axonal integrity and regeneration, but the post-transcriptional mechanisms regulating their development and reparative function remain poorly defined. Here, we investigated the isoform-specific roles of the RNA-binding protein Quaking (QKI), which is alternatively spliced into nuclear (QKI-5) and cytoplasmic (QKI-6 and QKI-7) variants, in governing human Schwann lineage progression. Using a human pluripotent stem cell-derived Schwann cell precursor (SCP) platform, we found that QKI-6 and QKI-7 are selectively upregulated during SCP-to-SC transition, whereas QKI depletion disrupts SCP viability, differentiation, and splicing fidelity. Transcriptomic and rMATS analysis identified more than 800 QKI-dependent splicing events, including disease-relevant isoform shifts in PLP1 and PMP22. Isoform-specific rescue and gain-of-function assays revealed that QKI-6 supports SCP expansion and mitotic progression, whereas QKI-7 promotes SC maturation and neurotrophic output. In a mouse sciatic nerve transection model, transplantation of QKI-7-overexpressing SCPs or SCs significantly enhanced axonal regeneration, remyelination, and motor recovery compared with unmodified or QKI-6-expressing counterparts. Histological analysis confirmed improved donor cell engraftment, myelin protein expression, and neurotrophin levels in QKI-7-modified grafts. These findings establish a sequential, isoform-dependent mechanism of Schwann lineage control and nominate QKI-7 as a candidate for engineering reparative glial cells with enhanced regenerative capacity. Isoform-targeted modulation of RNA-binding proteins may represent a strategy to overcome intrinsic limitations in glial cell therapy for peripheral nerve disorders.

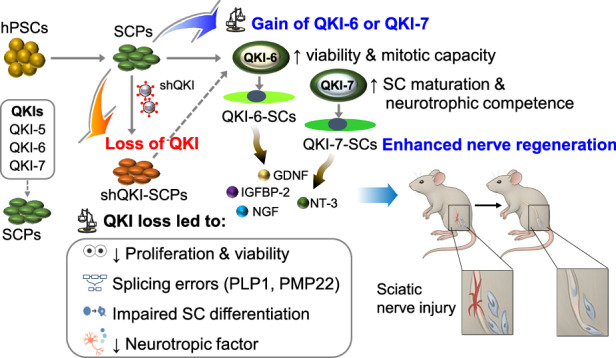

## Schwann cells: QKI isoforms regulate nerve repair

Peripheral nerve injuries often lead to long-term sensory and motor deficits, with limited treatment options. Schwann cells (SCs), crucial for nerve repair, originate from Schwann cell precursors (SCPs). This study explores the roles of Quaking (QKI) RNA-binding protein isoforms in SCP-to-SC differentiation. Using human pluripotent stem cells, researchers identified QKI-6 and QKI-7 as key regulators of SCP viability, proliferation, and neurotrophic output. QKI-7 notably enhances axonal regeneration both in vitro and in vivo. The study used lentiviral knockdown and overexpression techniques to assess isoform-specific functions, revealing that QKI-6 supports early SCP expansion, whereas QKI-7 promotes SC maturation and neurotrophic specialization. These findings suggest that QKI isoforms could be targeted for developing reparative glial cells, offering a promising strategy for peripheral nerve repair.

This summary was initially drafted using artificial intelligence, then revised and fact-checked by the author.

## Introduction

Peripheral nerve injuries result in long-term sensory and motor deficits and affect millions of patients annually, with limited therapeutic options beyond nerve grafting. Schwann cells (SCs), the myelinating glia of the peripheral nervous system (PNS), are essential for preserving axonal integrity, facilitating regeneration, and restoring functional recovery after injury^1^. These cells originate from Schwann cell precursors (SCPs), a transient neural crest-derived population that represents the earliest committed glial lineage in the PNS^2,3^. Owing to their proliferative potential and developmental plasticity, SCPs are increasingly recognized not only as an important intermediate during peripheral glial development but also as a scalable cellular source for regenerative therapies targeting peripheral nerve injuries and peripheral neuropathies.

Lineage progression from SCPs to mature SCs is orchestrated by extrinsic cues, including neuregulin-1 (NRG1), Notch, basic fibroblast growth factor, and transforming growth factor-β (TGF-β), which converge on intracellular pathways including PI3K/AKT and MAPK/ERK to regulate SCP survival, proliferation, and differentiation^4–7^. Although these pathways have been extensively characterized, they do not fully explain the precise temporal and spatial regulation of gene expression required for SC lineage specification, particularly under regenerative or pathological conditions. Despite increasing recognition of regulatory mechanisms beyond transcriptional control, the contribution of post-transcriptional regulation to the SCP-to-SC transition remains incompletely understood. In particular, how RNA-level regulatory processes contribute to the dynamic control of gene expression during SC differentiation and regeneration has not been fully elucidated.

RNA-binding proteins (RBPs) have a central role in post-transcriptional regulation by controlling alternative splicing, mRNA stability, localization, and translation^8^. Among these, the Quaking (QKI) family of RBPs, comprising three major isoforms (*QKI-5*, *QKI-6*, and *QKI-7*), has been implicated in neural development and glial maturation. These isoforms arise through alternative 3ʹ splicing and exhibit distinct subcellular localizations, with QKI-5 predominantly localized to the nucleus and QKI-6 and QKI-7 enriched in the cytoplasm^9,10^. In oligodendrocytes of the central nervous system (CNS), *QKI* regulates myelin gene expression and glial maturation by modulating alternative splicing and mRNA stability of key transcripts such as *MBP* and *PLP1* (refs. ^9–16^). In addition, QKI influences microRNA biogenesis, including repression of pri-miR-7 processing, thereby modulating downstream signaling pathways such as epidermal growth factor receptor (EGFR)/ERK^17^.

Despite these well-characterized roles in CNS myelination, the function of QKI isoforms in PNS glial cells remains poorly defined. Unlike oligodendrocytes, SCs ensheath individual axons, exhibit distinct developmental trajectories, and undergo rapid phenotypic remodeling following nerve injury. Moreover, the molecular composition and turnover dynamics of PNS myelin differ substantially from those of the CNS, raising the possibility that QKI isoforms may exert context-dependent functions across these compartments. Supporting this notion, QKI-6 and QKI-7 proteins are expressed in adult SCs and are downregulated in P0-deficient mice exhibiting demyelination, suggesting a role for QKI in maintaining PNS myelin integrity^18^. Consistent with this idea, dysregulation of alternative splicing mediated by RBPs has been linked to peripheral glial pathologies, including schwannoma genesis associated with NF2 splicing defects^19^, and aberrant QKI expression has been implicated in inherited demyelinating disorders such as Charcot–Marie–Tooth disease and Pelizaeus–Merzbacher disease^20,21^. Nevertheless, whether individual QKI isoforms exert distinct and isoform-specific functions during human SCP differentiation, SC maturation, and regenerative responses has not been systematically examined. Furthermore, the impact of QKI-mediated post-transcriptional regulation on neurotrophic factor expression and regenerative capacity in human Schwann lineage cells remains largely unexplored.

In this study, we used a stage-defined human pluripotent stem cell (hPSC) differentiation platform to investigate the isoform-specific roles of QKI-5, QKI-6, and QKI-7 during human SC lineage development. By combining isoform-specific perturbation with molecular, transcriptomic, and functional analyses, we demonstrate that QKI-6 and QKI-7 contribute to the regulation of SCP viability, proliferation, maturation, and neurotrophic output. Notably, QKI-7 enhances axonal regeneration both in vitro and in vivo. Together, these findings support a model in which QKI isoforms act as post-transcriptional regulators that fine-tune SC differentiation and regenerative capacity and highlight their potential utility as molecular targets for engineering reparative glial cells in peripheral nerve repair.

## Materials and methods

### Cell culture

Human embryonic stem cells (hESCs; H9 line; WiCell Research Institute, Madison, WI, USA), H9-derived SCPs (H9-SCPs), and H9-SCP-derived SCs (H9-SCP-SCs) were maintained under feeder-free conditions, as described previously^22^. hESCs were cultured on growth factor-reduced Matrigel-coated dishes (Corning, NY, USA) in mTeSR1 medium (STEMCELL Technologies, Vancouver, Canada) with daily medium changes.

SCPs were induced by replating hESCs on Matrigel-coated dishes and culturing in a chemically defined medium consisting of 1% N2 supplement, 2% B27 supplement (Thermo Fisher Scientific, Waltham, MA, USA), 0.005% bovine serum albumin (Sigma-Aldrich, St Louis, MO, USA), 2 mM GlutaMAX (Thermo Fisher Scientific), 0.11 mM β-mercaptoethanol (Sigma-Aldrich), 3 μM CHIR99021, and 20 μM SB431542 (Tocris Bioscience, Bristol, UK), prepared in a 1:1 mixture of Advanced DMEM/F12 and Neurobasal medium (Thermo Fisher Scientific). After 6 days of culture, the medium was replaced with neural induction medium supplemented with 100 ng/ml NRG1 (PeproTech, Rocky Hill, NJ, USA). Cells were dissociated using Accutase (Millipore, Billerica, MA, USA) and expanded for an additional 18 days.

Neural crest stem cells (NCSCs) were derived using the STEMdiff™ Neural Crest Differentiation Kit (STEMCELL Technologies), according to manufacturer’s instructions. Briefly, hESCs were dissociated with Accutase and seeded at a density of 2 × 10^5^ cells/cm² in mTeSR1 medium supplemented with 10 μM Y-27632 (ROCK inhibitor) for 24 h, followed by 6 days of culture in STEMdiff Neural Crest Differentiation Medium.

Differentiation of SCPs into SCs was performed in stages. Cells were first cultured in DMEM (Thermo Fisher Scientific) containing 0.2% fetal bovine serum (FBS; Thermo Fisher Scientific), 200 ng/ml NRG1, 5 μM forskolin (Sigma-Aldrich), 1 μM all-*trans*-retinoic acid (RA; Sigma-Aldrich), 100 ng/ml PDGF-BB (PeproTech), and 0.11 mM β-mercaptoethanol for 2 days. This was followed by 4 days in medium without RA, and then 4 additional days in medium containing only NRG1.

Primary human SCs (ScienCell Research Laboratories, Carlsbad, CA, USA) were cultured in Schwann cell medium (ScienCell), according to the supplier’s protocol. Human neonatal foreskin fibroblasts (CRL-2097; ATCC, Manassas, VA, USA) were maintained in minimum essential medium (Thermo Fisher Scientific) supplemented with 15% FBS and 0.11 mM β-mercaptoethanol.

### Reverse transcription and PCR

Total RNA was isolated using TRIzol™ Reagent (Thermo Fisher Scientific) and reverse-transcribed with the SuperScript™ VILO cDNA Synthesis Kit (Thermo Fisher Scientific). Quantitative real-time PCR (qPCR) was performed using Fast SYBR™ Green Master Mix (Thermo Fisher Scientific) on a 7500 Fast Real-Time PCR System (Applied Biosystems, Foster City, CA, USA). Endpoint PCR was conducted using HOT FIREPol® Blend Master Mix (Solis BioDyne, Tartu, Estonia). Primer sequences are provided in Supplementary Tables 1 and 2.

### Immunoblotting

Cells were lysed in RIPA buffer (Thermo Fisher Scientific), and protein concentrations were determined using the Pierce™ BCA Protein Assay Kit (Thermo Fisher Scientific). Equal amounts of protein (20 μg) were separated by SDS–PAGE, transferred to polyvinylidene fluoride membranes (Millipore), and blocked with 5% skimmed milk in TBS-T (0.05% Tween-20). Membranes were incubated overnight at 4 °C with primary antibodies, followed by horseradish peroxidase-conjugated secondary antibodies (Thermo Fisher Scientific) for 1 h at room temperature. Detection was performed using SuperSignal™ West Pico PLUS substrate (Thermo Fisher Scientific) and imaged with the Amersham™ Imager 600 (GE Healthcare). Antibodies are listed in Supplementary Table 3.

### Lentivirus preparation

*QKI* knockdown was achieved using a lentiviral vector system (*QKI* Human short hairpin RNA (shRNA) Lentiviral Plasmid Kit; OriGene, Rockville, MD, USA). Overexpression constructs for *QKI-6* and *QKI-7* were obtained from VectorBuilder (Chicago, IL, USA). Lentiviruses were produced by co-transfecting HEK293T cells with packaging plasmids using the 293 Expression Transfection Reagent (Excellgen, Rockville, MD, USA). Viral supernatants were collected at 48 h post-transfection, filtered (0.45 μm), and concentrated by ultracentrifugation at 100,000×*g* for 2 h at 4 °C. Viral pellets were resuspended in DMEM containing 0.5 M sucrose and stored at −80 °C.

### Immunocytometry

Cells were fixed in 4% formaldehyde in PBS for 10 min, permeabilized with 0.2% Triton X-100 containing 10% FBS and 1% bovine serum albumin for 1 h, and incubated with primary antibodies overnight at 4 °C. Following PBS washes, Alexa Fluor-conjugated secondary antibodies (Invitrogen, 1:500) were applied for 20 min at room temperature. Images were acquired using an Axio VertA.1 inverted microscope (Carl Zeiss, Oberkochen, Germany). Antibodies are listed in Supplementary Table 3.

### RNA-sequencing and alternative splicing analysis

GFP^+^ SCPs were isolated using FACSAria™ II (BD Biosciences, Franklin Lakes, NJ, USA). Total RNA was isolated with TRIzol, treated with DNase (Qiagen), and subjected to quality control before library construction using the TruSeq™ Stranded mRNA Kit (Illumina). Libraries were sequenced on a NovaSeq 6000 (Illumina) by Macrogen Inc. (Seoul, South Korea). Alternative splicing analysis was performed using rMATS v4.1.2, including events such as skipped exon (SE), alternative 3ʹ splice site, alternative 5ʹ splice site, mutually exclusive exons, and retained intron. Significant splicing events were defined by an average read count ≥10, false discovery rate ≤0.05, and absolute inclusion level difference (ΔPSI) > 0.1.

### Flow cytometry

For cell cycle analysis, cells were fixed in 70% ethanol at −20 °C for at least 2 h, washed with PBS, and stained with propidium iodide (PI; Sigma-Aldrich) and RNase A (Thermo Fisher Scientific). DNA content was analyzed using a BD Accuri™ C6 flow cytometer (BD Biosciences). Apoptosis was assessed using the Annexin V-APC/PI Apoptosis Detection Kit (BioLegend), following the manufacturer’s instructions, and subsequently analyzed by flow cytometry.

### Neurite outgrowth assay

SHSY-5Y neuronal cells (ATCC) were cultured in DMEM/F12 (Thermo Fisher Scientific) supplemented with 10% FBS and 1% penicillin/streptomycin. Cells were plated at 500 cells/cm² on Matrigel-coated 12-well plates, pre-differentiated with 1 μM RA for 1 day, and subsequently treated with conditioned media (CM) derived from SCPs or SCs. After 2 days, phase-contrast images were captured, and neurite lengths were quantified using AxioVision LE software (Carl Zeiss, version 4.8.2.0).

### ELISA

CM were filtered through a 0.2-μm membrane (Millipore) to eliminate cellular debris. Concentrations of human brain-derived neurotrophic factor (BDNF), glial cell line-derived neurotrophic factor (GDNF), and nerve growth factor (NGF) were measured using enzyme-linked immunosorbent assay (ELISA) kits (Abcam, Cambridge, MA, USA), following the manufacturer’s instructions. Absorbance was measured at 450 nm using a multiwell spectrophotometer (Molecular Devices, San Jose, CA, USA).

### Growth factor antibody profiling

Filtered CM samples were analyzed using the Human Growth Factor Antibody Array C1 (RayBiotech, Norcross, GA, USA; Cat no. AAH-GF-1), which detects 41 growth factors (amphiregulin, basic fibroblast growth factor/FGF-2, EGF, EGFR, FGF-4, FGF-6, FGF-7, granulocyte colony-stimulating factor (G-CSF), GDNF, granulocyte–macrophage colony-stimulating factor (GM-CSF), heparin-binding EGF-like growth factor, hepatocyte growth factor, insulin-like growth factor binding proteins (IGFBP1, IGFBP2, IGFBP3, IGFBP4, and IGFBP6), insulin-like growth factors (IGF-I and IGF-II), IGF-I soluble receptor, macrophage colony-stimulating factor (M-CSF), M-CSF receptor, β-NGF, neurotrophin-3 (NT-3), NT-4, platelet-derived growth factor receptors (PDGF receptor-α and receptor-β), PDGF-AA, PDGF-AB, PDGF-BB, placental growth factor (PLGF), stem cell factor (SCF), SCF receptor, TGFs (TGF-α, TGF-β1, TGF-β2, and TGF-β3), vascular endothelial growth factor A (VEGF-A), VEGF receptor 2, VEGF receptor 3, and VEGF-D). The array was processed according to the manufacturer’s instructions and visualized using the Amersham Imager 600 (GE Healthcare). Densitometric analysis was performed using ImageJ software.

### In vivo sciatic nerve injury and transplantation

All animal procedures were approved by the KRIBB Institutional Animal Care and Use Committee (KRIBB-AEC-18041). Eight-week-old male C57BL/6 mice (Dae Han BioLink, Chungju, South Korea) underwent sciatic nerve transection (SNT) and transplantation, as described previously^22^. A total of 1 × 10^5^ cells in 5 μl Matrigel were injected into the injury site. Transplanted cells included NCSCs, SCPs, QKI-7-overexpressing SCPs (QKI-7-SCPs), SCP-SCs, QKI-6-overexpressing SCP-derived SCs (QKI-6-SCP-SCs), and QKI-7-SCP-derived SCs (QKI-7-SCP-SCs). Mice were monitored for 8 weeks for functional recovery and histological evaluation.

### Rotarod test

Motor function was assessed using an accelerating rotarod (Daejong Instrument Industry, Seoul, South Korea). The rotation increased from 4 rpm to 40 rpm over 180 s, followed by a constant speed phase at 40 rpm for 120 s. Each mouse performed three trials with 10 min rest intervals. The average latency to fall was recorded.

### Immunohistochemistry

Mice were perfused with 4% paraformaldehyde, and sciatic nerves were post-fixed, cryoprotected in 30% sucrose, embedded in optimal cutting temperature (OCT) compound (Sakura Finetek, Torrance, CA, USA), and cryosectioned at 15 μm using a cryostat (Leica Microsystems, Wetzlar, Germany). Tissue sections were stained according to standard immunohistochemical protocols^22^. Antibodies are listed in Supplementary Table 3.

### Statistical analysis

Quantitative data are presented as mean ± standard deviations (s.d.), unless otherwise indicated. Two-group comparisons were analyzed using unpaired two-tailed Student’s *t* tests. For multiple group comparisons, one-way analysis of variance with Tukey’s post hoc test was applied. A *P*-value less than 0.01 was considered statistically significant.

## Results

### Stage-specific induction of QKI-6 and QKI-7 marks Schwann cell lineage progression

To define the molecular regulators of human SC lineage specification, we used a stepwise differentiation platform that guides hPSCs through NCSCs, SCPs, and terminally differentiated SCs (SCP-SCs). Transcriptomic profiling across these lineage-defined stages revealed the RNA-binding protein Quaking (*QKI*) as a candidate regulatory factor. Although QKI isoforms are known to coordinate myelination and glial maturation in the CNS^23,24^, their roles in peripheral glial development remain unexplored.

**Fig. 1:**
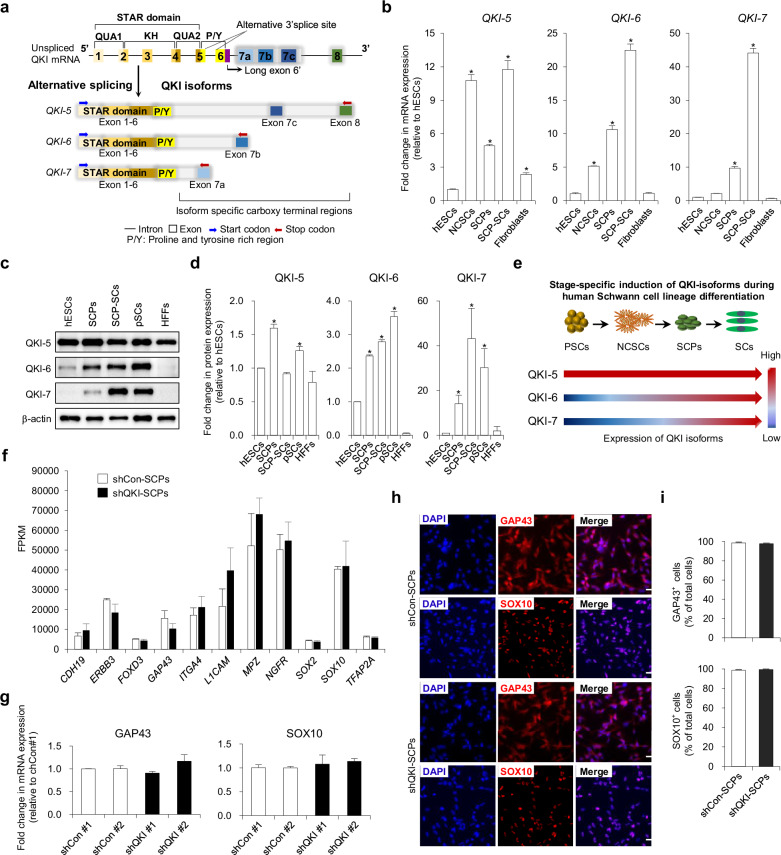
Stage-specific induction of QKI-6 and QKI-7 during Schwann cell lineage commitment from hPSCs. **a** Schematic representation of the three major *QKI* isoforms, *QKI-5* (predominantly nuclear), *QKI-6* (nuclear and cytoplasmic), and *QKI-7* (cytoplasmic), generated by alternative splicing. **b** qRT-PCR analysis of *QKI-5*, *QKI-6*, and *QKI-7* expression in undifferentiated human embryonic stem cells (hESCs), hESC-derived neural crest stem cells (NCSCs), hESC-derived Schwann cell precursors (SCPs), SCP-derived Schwann cells (SCP-SCs), and human foreskin fibroblasts (HFFs). Data are presented as mean ± s.d. (*n* = 3 independent biological replicates from independent differentiation experiments). Statistical significance was assessed using a two-tailed Student’s *t* test. *P < 0.01 (versus hESCs). **c** Immunoblot analysis of QKI isoforms in hESCs, SCPs, SCP-SCs, primary human Schwann cells (pSCs), and HFFs using isoform-specific antibodies. **d** Densitometric quantification of QKI protein levels from part **c**, normalized to β-actin and expressed relative to hESCs. Data are shown as mean ± s.d. (*n* = 3–5 independent biological replicates, depending on experimental availability). Statistical significance was assessed using a two-tailed Student’s *t* test. *P < 0.01 (versus hESCs). **e** Schematic summary of dynamic QKI isoform expression across distinct stages of Schwann cell differentiation. **f** RNA-sequencing analysis of SCPs transduced with either control short hairpin RNAs (shCon #1, #2) or *QKI*-targeting short hairpin RNAs (shQKI #1, #2), showing transcript levels (FPKM) of selected SCP markers. **g** Quantitative real-time PCR validation of *GAP43* and *SOX10* mRNA expression in shCon-transduced and shQKI-transduced SCPs (shQKI-SCPs). Expression was normalized to shCon#1. Data are presented as mean ± s.d. (*n* = 3 independent biological replicates). Statistical significance was assessed using a two-tailed Student’s *t* test. *P < 0.01. **h** Representative immunofluorescence images of GAP43 and SOX10 in shCon-SCPs and shQKI-SCPs. Nuclei were counterstained with 4′,6-diamidino-2-phenylindole (DAPI). Scale bar, 50 μm. **i** Quantification of GAP43⁺ and SOX10⁺ cell populations shown in part **h** Data are presented as mean ± s.d. (*n* = 3–5 independent biological replicates, depending on experimental availability). Statistical significance was assessed using a two-tailed Student’s *t* test. *P < 0.01.

The *QKI* gene encodes three major isoforms (QKI-5, QKI-6, and QKI-7) via alternative 3ʹ splicing, generating proteins with distinct C-terminal sequences that govern subcellular localization: QKI-5 is predominantly nuclear, QKI-6 distributes across nuclear and cytoplasmic compartments, and QKI-7 is restricted to the cytoplasm (Fig. 1a). To characterize the stage-specific expression of each isoform, we performed qPCR across undifferentiated hESCs, NCSCs, SCPs, SCP-SCs, and human foreskin fibroblasts as a somatic comparator. *QKI-6* and *QKI-7* transcripts were markedly upregulated at both the SCP and SCP-SC stages, with *QKI-7* exhibiting the most pronounced induction during SCP-to-SC maturation (Fig. 1b). By contrast, *QKI-5* expression remained largely unchanged throughout differentiation.

These transcript-level changes were mirrored at the protein level. Immunoblotting revealed strong induction of QKI-6 and QKI-7 in SCPs and SCP-SCs, with QKI-7 increasing most substantially during the SCP-SC transition (Fig. 1c). Densitometric analysis confirmed ~2.3-fold and ~3.6-fold increases in QKI-6 expression and dramatic ~30-fold to ~43-fold increases in QKI-7 in SCPs and SCP-SCs, respectively, relative to hESCs (Fig. 1d). A schematic summary illustrates the progressive and stage-specific induction of QKI isoforms along the SC lineage (Fig. 1e). These findings identify QKI-6 and QKI-7 as dynamically regulated isoforms that mark key transitions during human SC development, implicating them as candidate modulators of peripheral glial fate acquisition and maturation.

### QKI is dispensable for SCP identity but essential for maintaining mitogenic transcriptional programs

To define the functional requirement of QKI in SCPs, we performed lentiviral shRNA-mediated knockdown targeting of all three major isoforms (*QKI-5*, *QKI-6*, and *QKI-7*). Knockdown efficiency was validated by qPCR and immunoblotting, confirming robust depletion at both mRNA and protein levels (Supplementary Fig. 1a, b). Transcriptomic analysis of sorted GFP⁺ SCPs revealed that expression of canonical identity genes, including *CDH19*, *ERBB3*, *FOXD3*, *GAP43*, *ITGA4*, *L1CAM*, *MPZ*, *NGFR*, *SOX2*, *SOX10*, and *TFAP2A*, was largely preserved in QKI-depleted cells (Fig. 1f). These results were further corroborated by qRT-PCR for *GAP43* and *SOX10* (Fig. 1g) and by immunostaining and quantification of GAP43⁺ and SOX10⁺ cell populations (Fig. 1h,i), indicating that *QKI* is not essential for maintaining SCP identity at the transcriptional or protein level.

Despite preserved lineage identity, global transcriptome profiling identified widespread gene expression dysregulation in QKI-deficient SCPs. Replicates showed high concordance (Pearson *r* > 0.99; Supplementary Fig. 2a). Differential expression analysis identified 801 significantly altered transcripts, with 581 genes upregulated and 220 downregulated (Supplementary Fig. 2b). Gene ontology (GO) enrichment revealed strong associations with cell cycle regulation, mitosis, apoptosis, neurogenesis, angiogenesis, and sphingolipid metabolism (Supplementary Fig. 2c). KEGG pathway analysis further demonstrated coordinated downregulation of PI3K/Akt and MAPK signaling, ECM–receptor interaction, focal adhesion, and actin cytoskeleton regulation, pathways central to mitogenic signaling and SCP niche responsiveness (Supplementary Fig. 2d).

Notably, *QKI* knockdown resulted in significant suppression of G1/S and G2/M phase regulators including *CCNB1*, *CCNB2*, *CCNE1*, *CDK14*, *CDK2AP1*, *CDK19*, *CCND3*, and *CCNC* (Supplementary Fig. 2e). Conversely, transcripts linked to growth inhibition and cell cycle arrest, such as *CDKN1B*, *CDK18*, *CCNG2*, and *CCNDBP1*, were upregulated (Supplementary Fig. 2e), suggesting activation of intrinsic checkpoint mechanisms. This transcriptional profile indicates that although *QKI* is dispensable for maintaining SCP fate identity, it is essential for sustaining the mitogenic transcriptional network required for proliferation and progenitor maintenance.

### QKI deficiency compromises survival signaling and disrupts cell cycle progression in SCPs

**Fig. 2:**
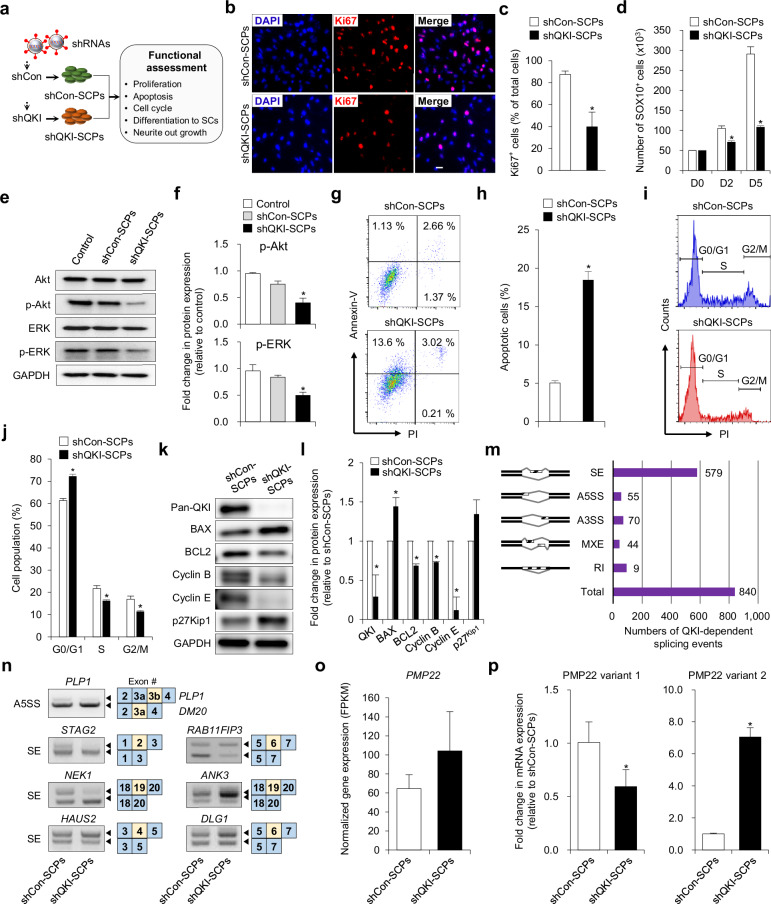
QKI knockdown disrupts mitogenic signaling, suppresses proliferation, and induces apoptosis in SCPs. **a** Schematic of the experimental workflow used to evaluate the functional impact of *QKI* knockdown in Schwann cell precursors (SCPs). **b** Representative immunofluorescence images showing Ki67 expression in shCon-SCPs and shQKI-SCPs. Nuclei were counterstained with 4′,6-diamidino-2-phenylindole (DAPI). Scale bar, 50 μm. **c** Quantification of Ki67^+^ nuclei in part **b** Data are presented as mean ± s.d. (*n* = 3 independent biological replicates). Statistical significance was assessed using a two-tailed Student’s *t* test. *P < 0.01 (versus shCon-SCPs). **d** Time-course quantification of SOX10⁺ SCPs at days 0, 2, and 5, based on GFP/SOX10 co-expression. Data are presented as mean ± s.d. (*n* = 4 independent biological replicates). Statistical significance was assessed using a two-tailed Student’s *t* test. *P < 0.01 (versus day 0). **e** Immunoblot analysis of phosphorylated and total AKT and ERK1/2 in control, shCon-SCPs, and shQKI-SCPs. GAPDH was used as a loading control. **f** Densitometric quantification of pAKT/AKT and pERK/ERK ratios from part **e**. Data are shown as mean ± s.d. (*n* = 3 independent biological replicates). Statistical significance was assessed using a two-tailed Student’s *t* test. *P < 0.01 (versus shCon-SCPs). **g** Representative flow cytometry plots showing Annexin V and propidium iodide (PI) staining in shCon-SCPs and shQKI-SCPs. **h** Quantification of Annexin V^+^ apoptotic cells from part **g** Data are presented as mean ± s.d. (*n* = 3 independent biological replicates). Statistical significance was assessed using a two-tailed Student’s *t* test. *P < 0.01 (versus shCon-SCPs). **i** Cell cycle analysis showing the distribut**i**on of SCPs across G0/G1, S, and G2/M phases by flow cytometry. **j** Quantitative summary of cell cycle phase distribution from part **i**. Data are presented as mean ± s.d. (*n* = 3 **i**ndependent biological replicates). Statistical significance was assessed using a two-tailed Student’s *t* test. *P < 0.01 (versus shCon-SCPs). **k** Immunoblot analysis of apoptosis-related proteins (BAX and BCL2), cell cycle regulators (Cyclin B1, Cyclin E, and p27^Kip1^), and Pan-QKI in shCon-SCPs and shQKI-SCPs. GAPDH served as a loading control. **l** Densitometric quantification of protein levels from part **k** Data are shown as mean ± s.d. (*n* = 3 independent biological replicates). Statistical significance was assessed using a two-tailed Student’s *t* test. *P < 0.01 (versus shCon-SCPs). **m** Classification and quantification of QKI-regulated alternative splicing events identified by rMATS, including skipped exons (SEs), alternative 5′ splice sites (A5SS), alternative 3′ splice sites (A3SS), mutually exclusive exons (MXEs), and retained introns (RIs). **n** Quantitative PCR (qPCR) validation of representative *QKI*-dependent alternative splicing events (A5SS and SE) in genes associated with cell cycle regulation. **o**, **p** RNA-sequencing analysis showing differential transcript levels (FPKM) of the *PMP22* gene in shCon-SCPs and shQKI-SCPs. **o** qPCR analysis of total *PMP22* expression in shCon-SCPs and shQKI-SCPs. **p** qPCR analysis of *PMP22* splice variants (variant 1 and variant 2), showing isoform imbalance consistent with splicing dysregulation characteristic of Charcot–Marie–Tooth disease type 1A (CMT1A). *GAPDH* was used as a normalization control. Data are shown as mean ± s.d. (*n* = 3 independent biological replicates). Statistical significance was assessed using a two-tailed Student’s *t* test. *P < 0.01 (versus shCon-SCPs). SC, Schwann cell; shRNA, short hairpin RNA.

To delineate the functional role of QKI in SCPs, we performed integrative phenotypic and molecular analyses following lentiviral-mediated knockdown of all three major isoforms (Fig. 2a). Immunofluorescence staining for Ki67 revealed a pronounced reduction in proliferating SCPs upon QKI depletion (Fig. 2b). Quantitative assessment confirmed a significant drop in the percentage of Ki67⁺ nuclei in QKI-deficient cultures (39.7 ± 13.3%) compared with controls (87.3 ± 3.1%, *P* < 0.01; Fig. 2c). In parallel, time-course analyses of SOX10⁺ SCPs demonstrated a progressive decline in cell numbers, suggesting impaired self-renewal capacity (Fig. 2d).

To investigate the underlying mechanisms, we examined phosphorylation of AKT and ERK1/2, key effectors of the PI3K/AKT and MAPK pathways critical for mitogenic signaling. QKI knockdown led to reduced pAKT and pERK levels (Fig. 2e), with densitometric quantification confirming significant suppression (Fig. 2f). These alterations were accompanied by increased Annexin V⁺ apoptotic cells as measured by flow cytometry (Fig. 2g,h) and a shift in cell cycle distribution, characterized by G_0_/G_1_ arrest and diminished S and G_2_/M fractions (Fig. 2i,j). At the protein level, QKI-deficient SCPs exhibited upregulation of the cyclin-dependent kinase inhibitor p27^Kip1^ and the pro-apoptotic factor BAX, alongside reductions in BCL2, Cyclin B1, and Cyclin E (Fig. 2k,l). These findings suggest that QKI is essential for maintaining SCP homeostasis by sustaining mitogenic signaling and cell cycle progression, and by preventing apoptotic entry, likely through post-transcriptional regulation of key mRNA targets involved in proliferation and survival.

### Loss of QKI alters alternative splicing programs governing SCP proliferation and glial integrity

Given the essential role of QKI in SCP proliferation and survival, we next examined whether it also regulates alternative splicing programs critical for SCP function. We performed replicate multivariate analysis of transcript splicing (rMATS) using RNA-sequencing data from control and QKI-deficient SCPs. *QKI* knockdown led to extensive post-transcriptional dysregulation, with 840 significant alternative splicing events identified (false discovery rate < 0.05, ΔPSI > 0.1), the majority of which (579/840; 68.9%) corresponded to SE events (Fig. 2m and Supplementary Fig. 3a). These splicing changes were consistent across replicates, with 264 exons exhibiting increased inclusion and 315 showing increased skipping, reflecting widespread and robust QKI-dependent regulation.

GO enrichment analysis of differentially spliced genes revealed strong associations with processes fundamental to SCP biology, including cell cycle progression, mitotic control, intracellular signaling, and transcriptional regulation (Supplementary Fig. 3b). A subset of 25 genes showed coordinated changes in both splicing and overall transcript levels (Supplementary Fig. 3c), indicating that QKI co-regulates mRNA abundance and isoform usage. These dual-regulated targets encompassed genes involved in neurodevelopment (*DAB1* and *ANK3*), cell adhesion (*COL11A2* and *COL12A1*), lipid metabolism (*SMPD1* and *ENPP2*), and vesicle-mediated transport (*PFN2* and *EGF*), implicating QKI in safeguarding the structural and functional integrity of glial progenitors.

To validate rMATS-identified events, we conducted isoform-specific RT-PCR on select targets. Aberrant exon inclusion or skipping was confirmed in SCP-relevant transcripts, including *STAG2*, *NEK1*, *HAUS2*, *RAB11FIP3*, *ANK3*, and *DLG1* (Fig. 2n), implicating QKI in the regulation of splicing networks involved in proliferation, cytoskeletal remodeling, and glial maturation. Furthermore, QKI depletion disrupted the isoform equilibrium of myelin-associated genes implicated in hereditary neuropathies. Notably, *PLP1* expression shifted toward the *DM20* isoform (Fig. 2n), a pattern associated with oligodendrocyte dysfunction and Pelizaeus–Merzbacher disease. Similarly, *PMP22* exhibited increased expression of variant 2 and reduced expression of variant 1 (Fig. 2o,p), recapitulating the splicing imbalance linked to Charcot–Marie–Tooth disease type 1A (CMT1A)^20^. Collectively, these findings position QKI as a pivotal regulator of alternative splicing fidelity in developing SCs, coordinating programs essential for cell cycle progression, neurodevelopment, and maintenance of glial identity.

### QKI is required for Schwann cell survival, terminal maturation, and neurotrophic function

**Fig. 3:**
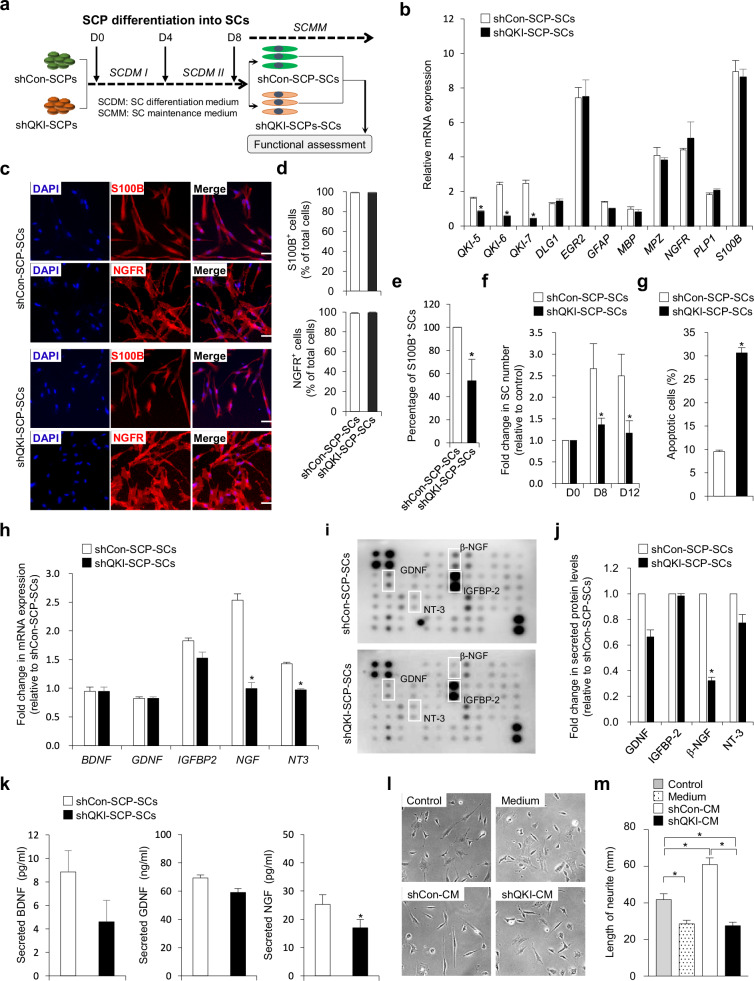
Loss of QKI impairs Schwann cell differentiation and neurotrophic factor production in SCP-derived SCs. **a** Schematic of the differentiation workflow used to assess the effects of QKI knockdown in Schwann cell precursor (SCP)-derived Schwann cells (SCP-SCs). **b** Quantitative PCR analysis of *QKI-5*, *QKI-6*, and *QKI-7* and SC marker genes (*DLG1*, *EGR2*, *GFAP*, *MBP*, *MPZ*, *NGFR*, *PLP1*, and *S100B*) in shCon-SCP-SCs and shQKI-SCP-SCs. Gene expression was normalized to *GAPDH*. Data are presented as mean ± s.d. (*n* = 3 independent biological replicates). Statistical significance was assessed using a two-tailed Student’s *t* test. *P < 0.01 (versus shCon-SCP-SCs). **c** Representative immunofluorescence images showing S100B and NGFR in shCon-SCP-SCs and shQKI-SCP-SCs. Nuclei were counterstained with 4′,6-diamidino-2-phenylindole (DAPI). Scale bar, 50 μm. **d** Quantification of S100B^+^ and NGFR^+^ cells shown in part **c** Data are presented as mean ± s.d. (*n* = 3 independent biological replicates). Statistical significance was assessed using a two-tailed Student’s *t* test. *P < 0.01 (versus shCon-SCP-SCs). **e** Differentiation efficiency of SCPs into S100B⁺ SCs in shCon and shQKI groups, based on GFP/S100B co-expression. Data are shown as mean ± s.d. (*n* = 3 independent biological replicates). Statistical significance was assessed using a two-tailed Student’s *t* test. *P < 0.01 (versus shCon-SCP-SCs). **f** Time-course analysis of SCP-SC expansion based on GFP expression at days 0, 8, and 12 of culture. Data are shown as mean ± s.d. (*n* = 4 independent biological replicates). Statistical significance was assessed using a two-tailed Student’s *t* test. *P < 0.01 (versus day 0). **g** Flow cytometric analysis of Annexin V⁺ apoptotic cells in shCon-SCP-SCs and shQKI-SCP-SCs. Data are presented as mean ± s.d. (*n* = 3 independent biological replicates). Statistical significance was assessed using a two-tailed Student’s *t* test. *P < 0.01 (versus shCon-SCP-SCs). **h** Quantitative PCR analysis of neurotrophic factor genes (*BDNF*, *GDNF*, *IGFBP2*, *NGF*, and *NT3*) in shCon-SCP-SCs and shQKI-SCP-SCs. Gene expression was normalized to *GAPDH*. Data are shown as mean ± s.d. (*n* = 3 independent biological replicates). Statistical significance was assessed using a two-tailed Student’s *t* test. *P < 0.01 (versus shCon-SCP-SCs). **i** Growth factor antibody array of conditioned media (CM) collected from shCon-SCP-SC and shQKI-SCP-SC cultures. Each spot represents a specific paracrine factor; signal intensity reflects relative abundance. **j** Densitometric quantification of glial cell line-derived neurotrophic factor (GDNF), insulin-like growth factor binding protein (IGFBP)-2, nerve growth factor (NGF), and neurotrophin (NT)-3 levels from part **i** Signal intensities were normalized to internal reference spots and analyzed using ImageJ. Data are presented as mean ± s.d. (*n* = 2 independent experiments; array data are presented for descriptive comparison). **k** Enzyme-linked immunosorbent assay quantification of brain-derived neurotrophic factor (BDNF), GDNF, and NGF levels in CM from shCon-SCP-SCs and shQKI-SCP-SCs. Values are expressed in pg/ml as mean ± s.d. (*n* = 3 independent biological replicates). Statistical significance was assessed using a two-tailed Student’s *t* test. *P < 0.01 (versus shCon-SCP-SCs). **l** Representative phase-contrast images of SH-SY5Y neuronal cells treated for 24 h with control medium (control), unconditioned medium (medium) or CM derived from shCon-SCP-SCs and shQKI-SCP-SCs (shCon-CM and shQKI-CM). **m** Quantification of average neurite length per neuron shown in part **l** based on measurements in 147–163 cells per group using AxioVision software. Data are presented as mean ± s.d. Statistical significance was assessed by one-way analysis of variance followed by Tukey’s post hoc multiple comparisons. *P < 0.01.

To determine whether QKI is required beyond the progenitor stage, we evaluated its role during terminal SC maturation. SCPs transduced with *QKI*-targeting shRNAs (shQKI) or control shRNAs (shCon) were differentiated into SCs (shQKI-SCP-SCs and shCon-SCP-SCs, respectively) and assessed for molecular identity, viability, and neurotrophic function (Fig. 3a). qPCR analysis revealed no significant differences in the expression of canonical Schwann cell markers, including *DLG1, EGR2, GFAP, MBP, MPZ, NGFR, PLP1*, and *S100B*, between control and QKI-deficient cells (Fig. 3b). Immunofluorescence confirmed comparable proportions of NGFR⁺ and S100B⁺ cells (Fig. 3c,d), indicating that QKI is not essential for initial lineage specification. Isoform-specific knockdown of QKI-5, QKI-6, and QKI-7 persisted throughout the differentiation process (Fig. 3b).

Despite intact marker expression, QKI-deficient SCPs showed reduced differentiation efficiency, as quantified by a significant decline in S100B⁺ Schwann cell yield (Fig. 3e). Time-course imaging demonstrated attenuated outgrowth and expansion in QKI-deficient cultures relative to controls (Fig. 3f). Flow cytometry confirmed elevated apoptosis in shQKI-SCP-SCs (Annexin V⁺/PI⁺: 30.6 ± 1.1%) compared with controls (9.5 ± 0.32%) (Fig. 3g), suggesting impaired survival.

To assess functional maturity, we examined neurotrophic factor production, a critical property of repair-competent Schwann cells. qPCR showed reduced expression of *NGF* and *NT3* in shQKI-SCP-SCs (Fig. 3h). Cytokine array profiling of CM revealed diminished secretion of key neurotrophic factors, including GDNF, NGF, and NT3 (Fig. 3i,j), findings corroborated by ELISA, which confirmed significant reductions in BDNF, GDNF, and NGF levels in QKI-deficient CM (Fig. 3k).

To evaluate neuroregenerative capacity in vitro, we used SH-SY5Y cells as a well-established human neuronal model that enables reproducible and quantitative assessment of neurite outgrowth in response to Schwann cell-derived trophic support under controlled conditions. SH-SY5Y neurons were exposed to CM collected from either shQKI-derived or shCon-derived SC cultures. Neuronal cultures treated with QKI-deficient CM exhibited significantly reduced neurite extension with those treated with control CM (27.6 ± 1.8 μm versus 61.0 ± 3.6 μm, respectively; *P* < 0.01) (Fig. 3l,m), indicating impaired neurotrophic support. Together, these results indicate that although QKI is not required for the acquisition of Schwann cell identity, it is essential for maintaining Schwann cell viability and expansion and for enabling the production of a neurotrophically active secretome, hallmarks of terminally mature, repair-competent Schwann cells.

### QKI-6 and QKI-7 exert isoform-specific effects on SCP survival, proliferation, and neurotrophic programming

**Fig. 4:**
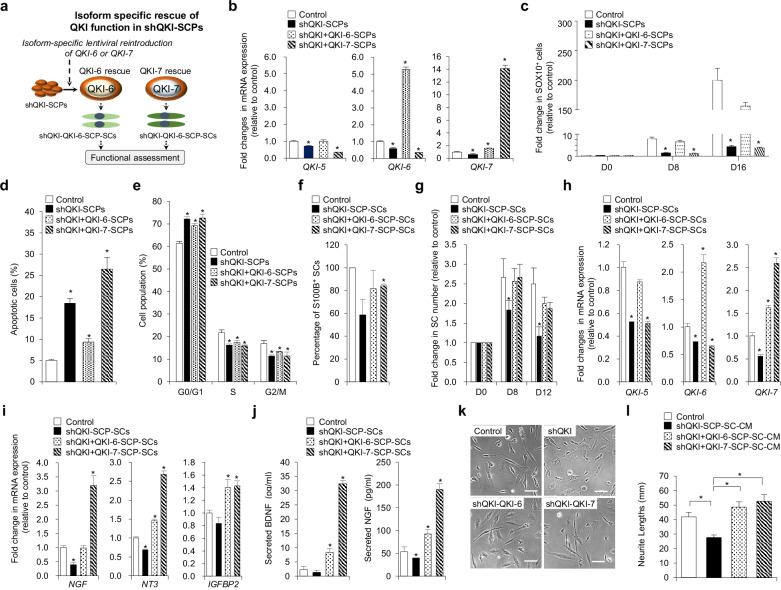
Isoform-specific rescue by QKI-6 and QKI-7 restores SCP identity, cell cycle progression, and survival. **a** Schematic of the experimental workflow to evaluate the rescue effects of *QKI-6* or *QKI-7* overexpression in *QKI*-deficient Schwann cell precursors (SCPs) and their derivative Schwann cells (SCs). **b** Quantitative PCR (qPCR) analysis of *QKI-5*, *QKI-6*, and *QKI-7* expression in unmodified SCPs (control), shQKI-SCPs, and shQKI-SCPs transduced with *QKI-6* (shQKI-QKI-6-SCPs) or *QKI-7* (shQKI-QKI-7-SCPs). Gene expression was normalized to *GAPDH*. Data are presented as mean ± s.d. (*n* = 3 independent biological replicates). Statistical significance was assessed using a two-tailed Student’s *t* test. *P < 0.01 (versus control). **c** Quantifi**c**ation of SOX10⁺ SCPs at days 0, 8, and 16 of culture in the indicated groups by immunocytochemistry. Data are shown as mean ± s.d. (*n* = 3 independent biological replicates). Statistical significance was assessed using a two-tailed Student’s *t* test. *P < 0.01 (versus control). **d** Percentage of Annexin V⁺ apoptotic cells in each group assessed by flow cytometry. Data are presented as mean ± s.d. (*n* = 3 independent biological replicates). Statistical significance was assessed using a two-tailed Student’s *t* test. *P < 0.01 (versus control). **e** Cell cycle distribution of SCPs across G0/G1, S, and G2/M phases analyzed by flow cytometry. Data are shown as mean ± s.d. (*n* = 3 independent biological replicates). Statistical significance was assessed using a two-tailed Student’s *t* test. *P < 0.01 (versus control). **f** Differentiation efficiency of control, shQKI-SCPs, shQKI-QKI-6-SCPs, and shQKI-QKI-7-SCPs into S100B⁺ SCs, quantified by GFP/S100B co-expression. Data are shown as mean ± s.d. (*n* = 3 independent biological replicates). Statistical significance was assessed using a two-tailed Student’s *t* test. *P < 0.01 (versus control). **g** Time-course analysis of SCP-SC expansion at days 8 and 12 based on GFP expression in the indicated groups. Data are presented as mean ± s.d. (*n* = 3 independent biological replicates). Statistical significance was assessed using a two-tailed Student’s *t* test. *P < 0.01 (versus control). **h** qPCR analysis of *QKI-5*, *QKI-6*, and *QKI-7* in the same groups as in part **f** Gene expression was normalized to *GAPDH*. Data are shown as mean ± s.d. (*n* = 3 independent biological replicates). Statistical significance was assessed using a two-tailed Student’s *t* test. *P < 0.01 (versus control). **i** qPCR analysis of neurotrophic factor genes (*NGF*, *NT3*, and *IGFBP2*) in the same groups as in part **f**. Gene expression was normalized to *GAPDH*. Data are shown as mean ± s.d. (*n* = 3 independent biological replicates). Statistical significance was assessed using a two-tailed Student’s *t* test. *P < 0.01 (versus control). **j** Enzyme-linked immunosorbent assay quantification of secreted brain-derived neurotrophic factor (BDNF) and nerve growth factor (NGF) levels in conditioned media (CM) from the same groups. Data are expressed in pg/ml as mean ± s.d. (*n* = 3 independent biological replicates). Statistical significance was assessed using a two-tailed Student’s *t* test. *P < 0.01 (versus control). **k** Representative phase-contrast images of SH-SY5Y neuronal cells treated for 24 h with CM from the groups in part **f**. **l** Quantification of average neurite length per neuron from part **k** based on measurements of 140–157 cel**l**s per group using AxioVision software. Data are mean ± s.d. Statistical significance was assessed by one-way analysis of variance followed by Tukey’s post hoc multiple comparisons. *P < 0.01.

To delineate isoform-specific functions of QKI during SCP regulation, we conducted rescue experiments by reintroducing either *QKI-6* or *QKI-7* into *QKI*-deficient SCPs, generating shQKI-QKI-6-SCPs and shQKI-QKI-7-SCPs, respectively (Fig. 4a). Ectopic expression of each isoform was confirmed by qPCR (Fig. 4b). Time-course analysis of SOX10⁺ cells showed that QKI-6 effectively restored and sustained SCP populations, consistent with re-established progenitor identity and proliferative capacity (Fig. 4c). By contrast, QKI-7 expression had minimal effect on SOX10⁺ cell abundance, suggesting limited capacity to support early progenitor maintenance.

Apoptosis and cell cycle analyses further underscored these functional differences. QKI-6 significantly reduced Annexin V⁺/PI⁺ apoptotic cells (9.35 ± 0.78%) relative to QKI-7 (26.5 ± 2.7%) (Fig. 4d) and rescued G2/M transition while alleviating G0/G1 arrest (Fig. 4e), indicating greater efficacy in supporting SCP survival and mitotic progression. Despite these early-stage differences, both isoforms restored the ability of QKI-deficient SCPs to generate SCs upon differentiation (Fig. 4f), as evidenced by progressive increases in viable SC counts in both rescue groups (Fig. 4g). Isoform overexpression remained stable throughout differentiation, as confirmed by qPCR (Fig. 4h).

Notably, isoform-specific divergence emerged in neurotrophic programming. Although both QKI-6 and QKI-7 enhanced IGFBP2 expression, QKI-7 more potently upregulated NGF and NT3 (Fig. 4i). ELISA measurements of CM revealed that QKI-7-rescued SCs secreted significantly higher levels of BDNF (32.4 ± 1.1 pg/ml) and NGF (190.2 ± 12.6 pg/ml) compared with QKI-6-rescued (BDNF: 8.2 ± 1.4 pg/ml; NGF: 92.6 ± 9.2 pg/ml) and QKI-deficient controls (BDNF: 2.3 ± 1.1 pg/ml; NGF: 53.7 ± 10.7 pg/ml) (Fig. 4j).

To assess the functional consequence of these changes, we treated SH-SY5Y neurons with CM from each rescue group. Neurons exposed to CM from either QKI-6 or QKI-7 rescue conditions showed significantly enhanced neurite outgrowth relative to QKI-deficient controls (Fig. 4k,l). Interestingly, neurite lengths were comparable between the two rescue groups, despite higher trophic factor levels in QKI-7 CM. This observation suggests either the presence of a saturation threshold for neurotrophic signaling or compensatory mechanisms mediated by QKI-6-dependent pathways. Together, these findings reveal distinct, stage-dependent roles for QKI isoforms in Schwann cell development.

### Gain-of-function analysis reveals isoform-specific roles of QKI-6 and QKI-7 in Schwann cell lineage expansion and neurotrophic maturation

**Fig. 5:**
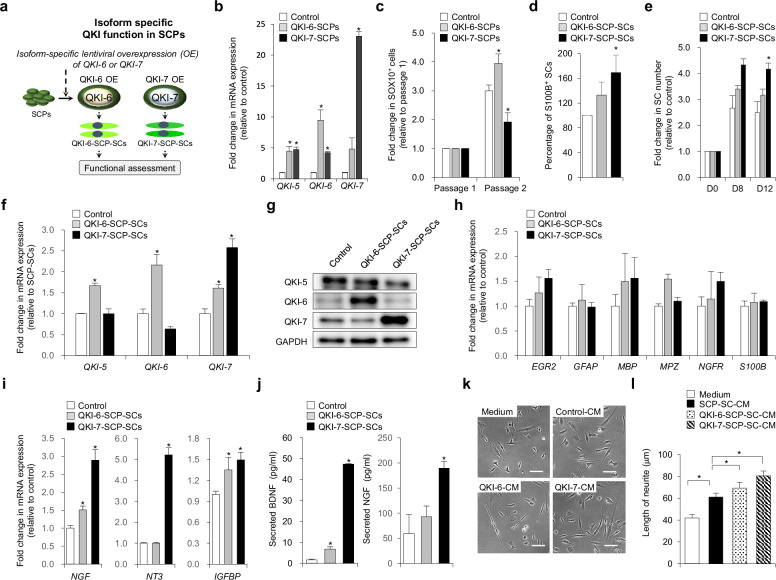
Ectopic expression of QKI-6 and QKI-7 differentially promotes SCP expansion, Schwann cell maturation, and neurotrophic support. **a** Schematic overview of the experimental workflow involving overexpression (OE) of *QKI-6* or *QKI-7* in Schwann cell precursors (SCPs), followed by functional analyses of *QKI-6*-overexpressing and *QKI-7*-overexpressing SCPs (QKI-6-SCPs and QKI-7-SCPs) and their differentiated Schwann cells (SCs) (QKI-6-SCP-SCs and QKI-7-SCP-SCs). **b** Quantitative PCR (qPCR) analysis of *QKI-5*, *QKI-6*, and *QKI-7* expression in unmodified SCPs (control), QKI-6-SCPs, and QKI-7-SCPs. Gene expression was normalized to *GAPDH*. Data are presented as mean ± s.d. (*n* = 3 independent biological replicates). Statistical significance was assessed using a two-tailed Student’s *t* test. *P < 0.01 (versus control). **c** Quantification of SOX10⁺ SCP expansion at passage 2 based on GFP/SOX10 co-expression. Data are presented as mean ± s.d. (*n* = 4 independent biological replicates). Statistical significance was assessed using a two-tailed Student’s *t* test. *P < 0.01 (versus control). **d** Differentiation efficiency of control, QKI-6-SCPs, and QKI-7-SCPs into S100B⁺ SCs, quantified by GFP/S100B co-expression. Data are shown as mean ± s.d. (*n* = 3 independent biological replicates). Statistical significance was assessed using a two-tailed Student’s *t* test. *P < 0.01 (versus control). **e** Time-course analysis of SCP-SC expansion at days 8 and 12 based on GFP expression. Data are present as mean ± s.d. (*n* = 4 independent biological replicates). Statistical significance was assessed using a two-tailed Student’s *t* test. *P < 0.01 (versus control). **f** qPCR analysis of *QKI-5*, *QKI-6*, and *QKI-7* in the same group as in part **d**. Gene expression was normalized to *GAPDH*. Data are presented as mean ± s.d. (*n* = 3 independent biological replicates). Statistical significance was assessed using a two-tailed Student’s *t* test. *P < 0.01 (versus control). **g** Immunoblot analysis of QKI isoforms in the same group. GAPDH was used as a loading control. **h** qPCR analysis of SC marker genes (*S100B*, *NGFR*, *MPZ*, *EGR2*, *MBP*, and *GFAP*) in the same group. Gene expression was normalized to *GAPDH*. Data are presented as mean ± s.d. (*n* = 3 independent biological replicates). Statistical significance was assessed using a two-tailed Student’s *t* test. *P < 0.01 (versus control). **i** qPCR analysis of neurotrophic factor genes (*NGF*, *NT3*, and *IGFBP2*) in the same group. Gene expression was normalized to *GAPDH*. Data are shown as mean ± s.d. (*n* = 3 independent biological replicates). Statistical significance was assessed using a two-tailed Student’s *t* test. *P < 0.01 (versus control). **j** Enzyme-linked immunosorbent assay quantification of secreted brain-derived neurotrophic factor (BDNF) and nerve growth factor (NGF) levels in conditioned media (CM) from the same groups. Data are presented in pg/ml as mean ± s.d. (*n* = 3 independent biological replicates). Statistical significance was assessed using a two-tailed Student’s *t* test. *P < 0.01 (versus control). **k** Representative phase-contrast images of SH-SY5Y neuronal cells cultured for 24 h with unconditioned medium (medium), CM from control SCP-SCs (control-CM), QKI-6-SCP-SCs (QKI-6-CM), or QKI-7-SCP-SCs (QKI-7-CM). **l** Quantification of average neurite length per neuron from part **k** based on measurements of 135–167 cells per group using AxioVision software. Data are presented as mean ± s.d. Statistical significance was assessed by one-way analysis of variance followed by Tukey’s post hoc multiple comparisons. *P < 0.01.

To delineate the physiological contributions of individual QKI isoforms in Schwann cell development, we conducted gain-of-function experiments by ectopically expressing *QKI-6* or *QKI-7* in wild-type SCPs (Fig. 5a). Successful isoform-specific overexpression was validated by qPCR (Fig. 5b). Global transcriptomic profiling via RNA-sequencing demonstrated strong inter-replicate concordance across experimental groups (Supplementary Fig. 4a). Differential expression analysis revealed 808 genes significantly altered by *QKI-6* or *QKI-7* overexpression (Supplementary Fig. 4b), whereas expression of canonical SCP identity genes, such as *ERBB3*, *FOXD3*, *GAP43*, *ITGA4*, *MPZ*, *SOX2*, *SOX10*, and *TFAP2A*, remained stable (Supplementary Fig. 4c), confirming preservation of SCP lineage fidelity.

GO and KEGG pathway enrichment analyses of differentially expressed genes indicated enrichment for biological processes such as cell adhesion and axon guidance (Supplementary Fig. 4d), suggesting that QKI isoforms influence cytoskeletal remodeling and environmental responsiveness. Both QKI-6 and QKI-7 increase expression of key mitotic regulators including *CCNB1*, *CCNB2*, *CCNE1*, *CDK14*, *CDK2AP*1, *CCND2*, and *CCNC* (Supplementary Fig. 4e), pointing to enhanced mitogenic activity.

At the cellular level, QKI-6 overexpression significantly expanded the SOX10⁺ SCP pool over serial passages, indicating its role in sustaining progenitor proliferation (Fig. 5c). By contrast, QKI-7 had a minimal impact on SCP maintenance, consistent with its limited effect observed in the rescue model. Upon induction of differentiation, both isoforms increased SC output relative to controls (Fig. 5d), although QKI-7 overexpression yielded a significantly higher number of viable SCs than QKI-6. Longitudinal analysis showed that QKI-7 supported sustained SC expansion, suggesting enhanced survival and maturation capacity (Fig. 5e). Isoform expression was stably maintained during the differentiation process (Fig. 5f, g), and key SC lineage markers, including *S100B*, *NGFR*, *MPZ*, *EGR2*, *MBP*, and *GFAP*, remained comparable across groups (Fig. 5h).

Transcriptomic analysis of neurotrophic gene programs revealed that QKI-7 more strongly induced expression of *NGF*, *NT3*, and *IGFBP2* compared with QKI-6 (Fig. 5i). CM from QKI-6-overexpressing and QKI-7-overexpressing SCs shared upregulation of regenerative factors such as G-CSF, GDNF, GM-CSF, β-NGF, NT-3, NT-4, PDGF isoforms, PLGF, SCF, VEGF-D, multiple IGFBPs (IGFBP2, IGFBP3, IGFBP4, and IGFBP6), and TGF isoforms (TGF-β1 and TGF-β3), as well as receptors including IGF-1R, M-CSFR, and VEGFR-3 (Supplementary Fig. 5a,b). Notably, only QKI-7-CM exhibited elevated levels of FGF-4, FGF-6, IGF-1, M-CSF, and TGF-α, indicating a broader and more specialized neurotrophic secretome.

ELISA confirmed higher secretion of BDNF and NGF by QKI-7-overexpressing SCs (BDNF: 47.3 ± 0.37 pg/ml and NGF: 189.6 ± 13.6 pg/ml) compared with QKI-6-overexpressing cells (BDNF: 6.7 ± 1.2 pg/ml and NGF: 93.2 ± 21.2 pg/ml) and controls (BDNF: 1.6 ± 0.35 pg/ml and NGF: 59.6 ± 37.7 pg/ml) (Fig. 5j). Functionally, CM from QKI-7-overexpressing SCs elicited significantly longer neurite outgrowth in SH-SY5Y neurons than CM from either QKI-6-overexpressing or control SCs (Fig. 5k,l). This outcome differed from the QKI-deficient rescue setting, in which both isoforms restored neurite extension to comparable levels (Fig. 4k,l), suggesting that the full neuroregenerative potential of QKI-7 may depend on intact endogenous QKI networks or specific transcriptional contexts. Together, these data highlight that QKI-6 predominantly supports SCP proliferation and early expansion, whereas QKI-7 drives terminal SC maturation and neurotrophic specialization.

### QKI-7 overexpression enhances Schwann cell-mediated peripheral nerve regeneration in vivo

**Fig. 6:**
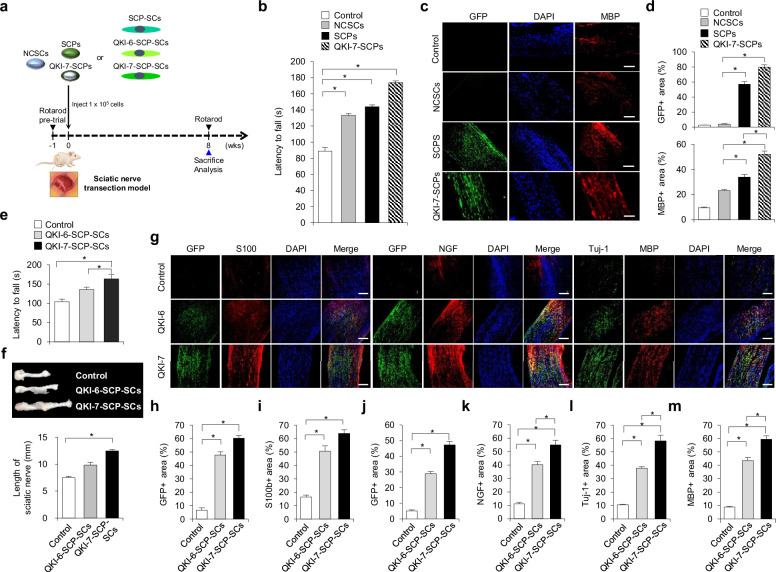
QKI-7-overexpressing Schwann cells promote enhanced peripheral nerve regeneration following sciatic nerve injury in vivo. **a** Schematic of the in vivo experimental design using a sciatic nerve transection (SNT) mouse model. GFP-labeled neural crest stem cells (NCSCs), Schwann cell precursors (SCPs), or SCP-Schwann cells (SCs) were transplanted into the injury site. Motor function was assessed at defined time points using the rotarod test, and tissues were collected for analysis 8 weeks post-transplantation. **b** Motor coordination scores, measured as latency to fall, were evaluated before injury and at 8 weeks post-SNT in mice receiving PBS (control), NCSCs, SCPs, or QKI-7-SCPs. The rotarod protocol consisted of an acceleration phase (4–40 rpm over 3 min) followed by a constant-speed phase (40 rpm for 2 min). Scores represent the average latency to fall from three trials per mouse with 10-min intertrial intervals. Data are presented as mean ± s.d. (*n* = 3 animals per group). Statistical significance was assessed by one-way analysis of variance (ANOVA) followed by Tukey’s post hoc multiple comparisons. *P < 0.01. **c** Representative immunofluorescence images of longitudinal sciatic nerve sections stained for myelin basic protein (MBP) at 8 weeks post-transplantation. Scale bar, 200 μm. **d** Quantification of regenerative indices within the graft site. Top: GFP⁺ area representing donor cell engraftment. Bottom: MBP⁺ area representing myelination. Data are shown as mean ± s.d. (*n* = 3–6 animals per group, depending on experimental availability). Statistical significance was assessed by one-way ANOVA followed by Tukey’s post hoc multiple comparisons. *P < 0.01. **e** Motor performance scores at baseline and 8 weeks post-SNT in mice transplanted with SCP-SCs (control), QKI-6-SCP-SCs, or QKI-7-SCP-SCs, measured using the same rotarod protocol as in part **b**. Data are presented as mean ± s.d. (*n* = 3 animals per group). Statistical significance was assessed by one-way ANOVA followed by Tukey’s post hoc multiple comparisons. *P < 0.01. **f** Representative image (top) and quantification (bottom) of regenerated sciatic nerve length at 8 weeks post-transplantation. Regenerated length (mm) across the graft site reflects axonal outgrowth. Data are presented as mean ± s.d. (*n* = 3 animals per group). Statistical significance was determined by one-way ANOVA followed by post hoc multiple comparisons. *P < 0.01. **g** Representative immunofluorescence images of sciatic nerve sections stained for S100B (myelinating SCs, top), nerve growth factor (NGF) (neurotrophic factor, middle), and Tuj1/MBP (neuronal and myelin markers, bottom). Scale bar, 200 μm. **h**–**m** Quantification of regenerative markers in the transplanted nerve segment. GFP⁺ area (donor cell engraftment) (parts **h** and **j**); S100B⁺ area (myelinating SCs) (part **i**); NGF⁺ area (neurotrophic support) (part **k**); Tuj1⁺ and MBP⁺ areas (parts **l** and **m**). Data are presented as mean ± s.d. (*n* = 3 animals per group). Statistical significance was assessed by one-way ANOVA followed by Tukey’s post hoc multiple comparisons. *P < 0.01. All behavioral assessments and histological analyses shown in this figure were performed at 8 weeks post-transplantation. DAPI, 4′,6-diamidino-2-phenylindole.

To evaluate the translational potential of QKI isoforms in promoting nerve repair, we assessed their regenerative efficacy using a murine SNT model with transplantation of engineered human glial lineage cells (Fig. 6a). Two cohorts were generated for comparison: a progenitor-stage group comprising NCSCs, SCPs, and QKI-7-SCPs, and a terminally differentiated group composed of QKI-6-SCP-SCs and QKI-7-SCP-SCs.

Motor coordination was assessed using the rotarod test at 8 weeks after cell grafting. At this post-graft assessment, all progenitor-derived grafts significantly improved motor performance compared with PBS-treated, post-injury control animals. Among these groups, QKI-7-SCPs-transplanted mice exhibited the greatest improvement, as indicated by a significantly increased latency to fall relative to both NCSC-transplanted and unmodified SCP-transplanted mice (control: 88.9 ± 4.56 s, NCSCs: 133.2 ± 2.45 s, SCPs: 144.0 ± 2.23 s, QKI-7-SCPs: 173.8 ± 2.29 s; Fig. 6b). Immunohistochemical analyses of longitudinal sciatic nerve sections performed at 8 weeks post-transplantation revealed robust donor cell engraftment and myelin regeneration in the SCP and QKI-7-SCP groups, as evidenced by extensive GFP⁺ cell distribution and MBP⁺ myelin formation in the SCP and QKI-7-SCP groups (Fig. 6c,d). Quantitative analysis demonstrated significantly larger myelinated regions in QKI-7-SCP recipients compared with other progenitor-derived grafts.

In the Schwann cell transplantation cohort, both QKI-6-SCP-SCs and QKI-7-SCP-SCs significantly enhanced motor recovery at 8 weeks post-graft relative to unmodified SCP-SCs, with QKI-7-SCP-SCs conferring the greatest functional benefit (control: 86.9 ± 0.8 s, QKI-6-SCP-SCs: 149.2 ± 4.4 s, QKI-7-SCP-SCs: 195.0 ± 2.86 s; Fig. 6e). These improvements were corroborated by morphometric analyses conducted at the same post-transplantation time point, which demonstrated that QKI-7-SCP-SC grafts generated the longest regenerated nerve bridges (control: 7.46 ± 0.26 mm, QKI-6-SCP-SCs: 9.84 ± 0.51 mm, QKI-7-SCP-SCs: 12.48 ± 0.27 mm; Fig. 6f). Consistent with these findings, immunohistochemical evaluation at 8 weeks post-graft confirmed widespread donor cell engraftment across all transplanted groups, as indicated by GFP⁺ cell distribution (Fig. 6g,h,j). Notably, QKI-7-SCP-SC recipients displayed the largest population of S100B⁺ Schwann cells (Fig. 6g,i) and the highest NGF⁺ immunoreactivity within the regenerative nerve segment (Fig. 6g,k), in agreement with the enhanced neurotrophic factor expression observed in vitro. Moreover, axonal regeneration was most pronounced in the QKI-7-SCP-SC group, as evidenced by expanded Tuj1⁺ axonal and MBP⁺ myelinated fiber domains compared with other experimental conditions (Fig. 6g, l, m).

Collectively, these results demonstrate that QKI-7 enhances post-transplantation functional recovery and tissue regeneration in vivo, primarily by promoting terminal Schwann cell maturation and neurotrophic competence. Although QKI-7 exerted minimal effects on early progenitor expansion, its pronounced impact on post-engraftment outcomes highlights a stage-specific role in driving regenerative efficacy. Together with the complementary effects of QKI-6 on early SCP survival and expansion, these findings support a dual-function model in which QKI isoforms coordinately regulate Schwann cell-mediated peripheral nerve repair.

## Discussion

This study identifies QKI-6 and QKI-7 as isoform-specific, non-redundant regulators of human SCP proliferation, Schwann cell (SC) maturation, and peripheral nerve regeneration. Using a stepwise hPSC differentiation system, we show that QKI-6 and QKI-7, but not the nuclear isoform QKI-5, are selectively induced during the SCP-to-SC transition and perform temporally distinct functions. QKI-6 supports early SCP expansion and survival, whereas QKI-7 promotes terminal differentiation and activates a trophically enriched secretome. These findings uncover a previously unappreciated layer of QKI-mediated regulation specific to the PNS, extending beyond its established roles in CNS myelination.

In the CNS, QKI isoforms are compartmentalized to support stage-specific functions in glial development: QKI-5 mediates nuclear RNA splicing and export, whereas cytoplasmic QKI-6 and QKI-7 regulate mRNA translation and stability, including key myelin transcripts such as MAG and MBP^12,14,16,25–27^. However, their roles in human peripheral glia have remained largely undefined. Here, we demonstrate that QKI-6 and QKI-7 are dynamically induced during peripheral glial differentiation and execute distinct but complementary functions essential for SCP homeostasis and SC specialization.

Loss-of-function studies revealed that QKI is essential for maintaining SCP mitogenic transcriptional programs. QKI knockdown led to downregulation of core G1/S and G2/M regulators (*CCNE1*, *CCNB1*, and *CDK14*) and concurrent upregulation of cell cycle inhibitors such as *CDKN1B* (p27^Kip1^), a known QKI target^28,29^, culminating in cell cycle arrest, reduced Ki67⁺ proliferation, and increased apoptosis. These effects were accompanied by suppression of PI3K/AKT and MAPK pathways, canonical mitogenic axes, indicating mitogenic collapse. These findings are consistent with the essential role of QKI in progenitor stability, previously described in oligodendrocyte precursors and glioma cells^28,30,31^.

In parallel, transcriptome-wide splicing analysis identified more than 800 QKI-regulated alternative splicing events in SCPs, many affecting genes involved in cytoskeletal remodeling, intracellular transport, and neurodevelopment. Notably, we observed pathogenic isoform shifts in *PLP1* and *PMP22*, genes implicated in Pelizaeus–Merzbacher disease^21,32,33^ and CMT1A^20,34^, respectively. These data implicate QKI as a molecular gatekeeper of peripheral glial transcriptome fidelity, preventing the emergence of disease-associated splicing variants during lineage progression.

Functionally, QKI-deficient SCs retained lineage markers but failed to sustain neurotrophic factor production and exhibited reduced survival and impaired support for neurite outgrowth. Isoform-specific rescue clarified this dichotomy: QKI-6 restored proliferation and mitigated apoptosis^16,30,35,36^, whereas QKI-7 reinstated NT gene expression but had limited impact on mitosis^9,16,18,24,28,37^. These findings suggest a functional division of labor, in which QKI-6 governs progenitor homeostasis and QKI-7 modulates terminal differentiation and secretome maturation.

Consistent with this model, overexpression of QKI-6 in wild-type SCPs enhanced their proliferative capacity without altering lineage identity, whereas QKI-7 accelerated Schwann cell differentiation and drove the emergence of a regenerative trophic profile. Proteomic analyses revealed that both isoform groups secreted key neurotrophic and pro-regenerative mediators, including NGF, NT-3, GDNF, IGFBPs, PDGFs, and TGF-βs^38–41^. However, QKI-7 uniquely increased the secretion of factors such as FGF-4, FGF-6, IGF-1, M-CSF, and TGF-α, which are known to support Schwann cell maturation, macrophage-mediated remodeling, and extracellular matrix reorganization^42–45^. Importantly, CM from QKI-7-overexpressing SCs induced greater neurite outgrowth in SH-SY5Y neurons than either QKI-6 or controls, confirming its superior neuroregenerative potential. Notably, this enhanced neurotrophic activity was not recapitulated in QKI-deficient cells rescued with QKI-7, suggesting that its full functional output may depend on the presence of intact QKI-dependent networks or cooperating RNA-binding proteins. By contrast, QKI-6 appeared to confer broader transcriptomic stabilization, enabling partial functional restoration even in the absence of endogenous QKI. These observations highlight the context dependence of isoform activity and the non-redundant nature of QKI-6 and QKI-7 functions.

In vivo, transplantation of QKI-6-overexpressing or QKI-7-overexpressing SCP-SCs into a SNT model significantly enhanced motor recovery and nerve regeneration. Although QKI-6 promoted progenitor survival and engraftment, QKI-7 conferred superior regenerative benefits, including greater S100B⁺ Schwann cell repopulation, increased NGF⁺ trophic zone size, and more extensive MBP⁺ and Tuj1⁺ axonal regrowth. Notably, QKI-7-SCP-SCs outperformed both NCSCs and unmodified SCPs despite comparable proliferation, suggesting that QKI-7 accelerates the acquisition of a trophically competent Schwann cell phenotype post-engraftment. These findings support a bifunctional model wherein QKI-6 ensures progenitor pool integrity, whereas QKI-7 orchestrates terminal differentiation and neurotrophic specialization.

This work offers key translational insights. Current strategies for peripheral nerve repair, including autologous nerve grafts, mesenchymal stromal cells, and partially specified precursors, are hindered by poor lineage fidelity, limited trophic output, or inconsistent engraftment^46–50^. By contrast, QKI isoform-guided engineering enables the generation of lineage-stable, reparative SC-like cells through precise post-transcriptional modulation. Unlike transcription factor-based reprogramming, which may destabilize lineage identity, QKI-targeted approaches preserve fate while enhancing function.

In conclusion, our findings establish QKI-6 and QKI-7 as sequential regulators of human Schwann cell lineage development. Acting in a temporally coordinated manner, QKI-6 supports progenitor expansion and transcriptomic stability, whereas QKI-7 drives neurotrophic programming and regenerative function. Their isoform-specific, context-dependent actions present a powerful paradigm for glial cell engineering and nominate QKI modulation as a clinically actionable strategy for peripheral nerve repair. Future studies in chronic injury models, inflammatory neuropathies, and large-gap reconstructions will be instrumental in validating the therapeutic durability and scalability of this approach.

## Supplementary information

Supplementary Information

## Data Availability

All data that support the findings of this study are available from the corresponding author upon reasonable request.
