# Supplementary material for: Isoform-specific roles of QKI-6 and QKI-7 direct Schwann cell lineage progression and enhance peripheral nerve regeneration
Source: Exp Mol Med. 2026 May 1;58(5):1449–63. doi: 10.1038/s12276-026-01708-0 (PMC13234107; doi:10.1038/s12276-026-01708-0)
Supplement: Supplementary file 1 — Supplementary Information [file 12276_2026_1708_MOESM1_ESM.pdf]

Supplementary Information

The Supplementary Information includes 5 figures and 3 tables

Supplementary Fig. 1

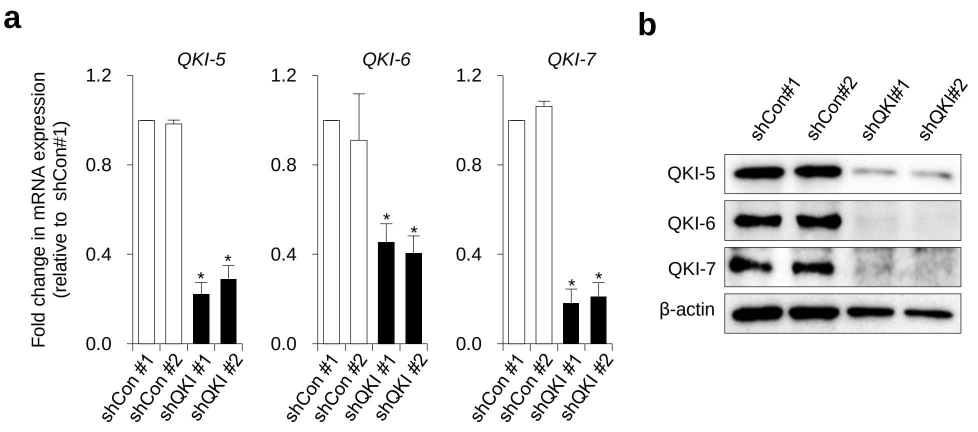

Supplementary Fig. 1 Efficient knockdown of QKI isoforms at the mRNA and protein levels in SCPs. a Quantitative real-time PCR (qPCR) analysis showing reduced expression of *QKI-5*, *QKI-6*, and *QKI-7* in SCPs transduced with *QKI*-targeting shRNAs (shQKI #1 and #2) compared with non-targeting controls (shCon #1 and #2). Gene expression was normalized to *GAPDH*. Data are presented as mean ± SD (n = 3 independent biological replicates). Statistical significance was assessed using a two-tailed Student's t-test. *p < 0.01 (*vs.* shCon). **b** Immunoblot analysis confirming isoform-specific reduction of QKI proteins following lentiviral shRNA transduction. β-actin was used as a loading control.

Supplementary Fig. 2

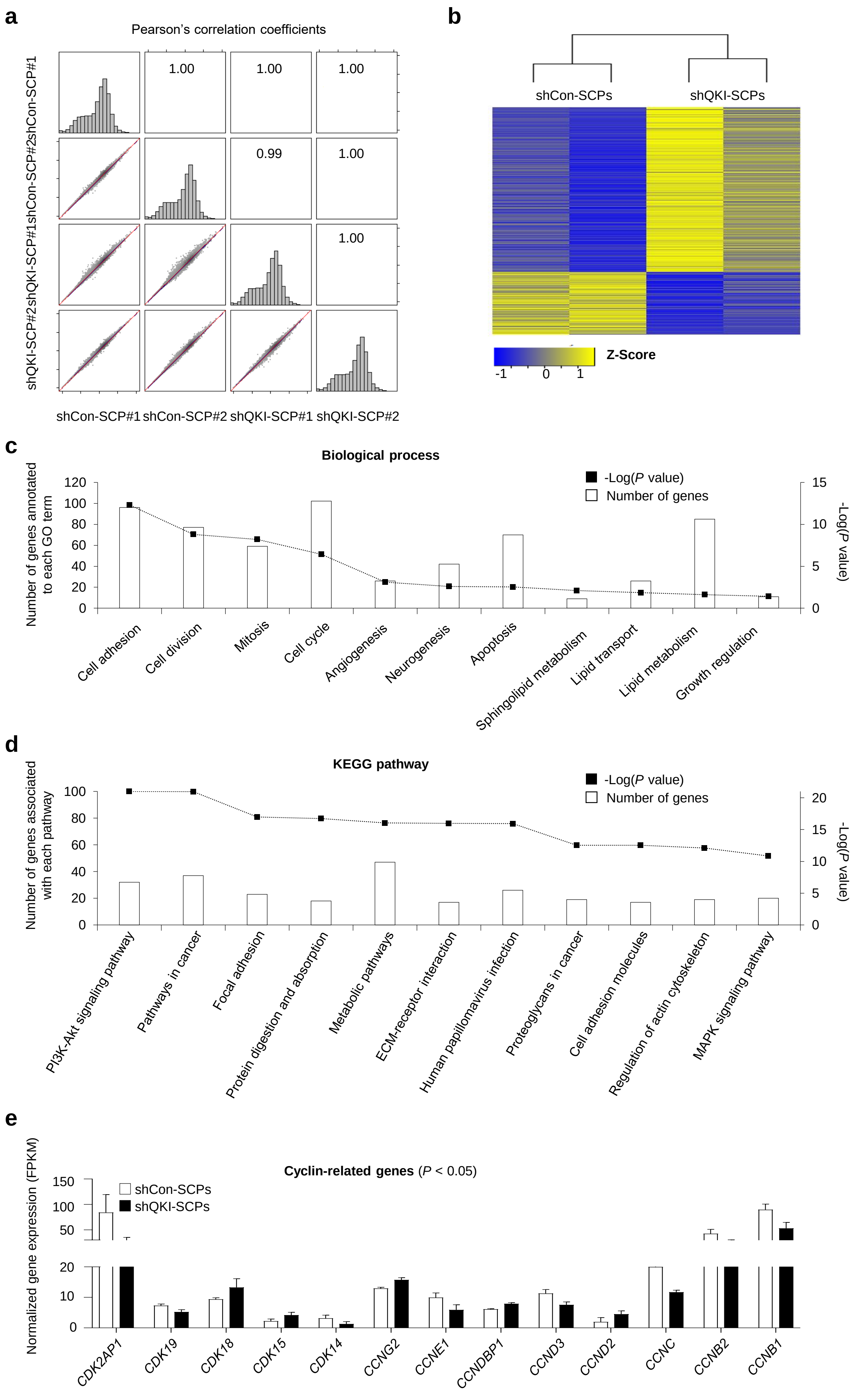

Supplementary Fig. 2 Transcriptomic profiling reveals QKI-dependent regulation of cell cycle gene expression in SCPs. a Pairwise Pearson correlation plots comparing global transcriptomic profiles of sh-Con- and shQKI-transduced SCPs (shQKI-SCPs). Correlations were calculated for genes with counts per million (CPM) > 10 following median normalization. **b** Hierarchical clustering heatmap of 801 differentially expressed genes (DEGs; fold change > 2, $p < 0.05$), revealing distinct transcriptional profiles between groups. **c** Gene Ontology (GO) enrichment analysis of significantly upregulated and downregulated DEGs, highlighting the Top 10 enriched biological processes. **d** KEGG pathway enrichment analysis of DEGs, illustrating major signaling and regulatory pathways affected by *QKI* knockdown. Bar lengths indicates the number of enriched genes per pathway. **e** FPKM values of representative cyclin genes associated with G1/S and G2/M transitions, demonstrating downregulation of proliferative regulators in QKI-deficient SCPs.

Supplementary Fig. 3

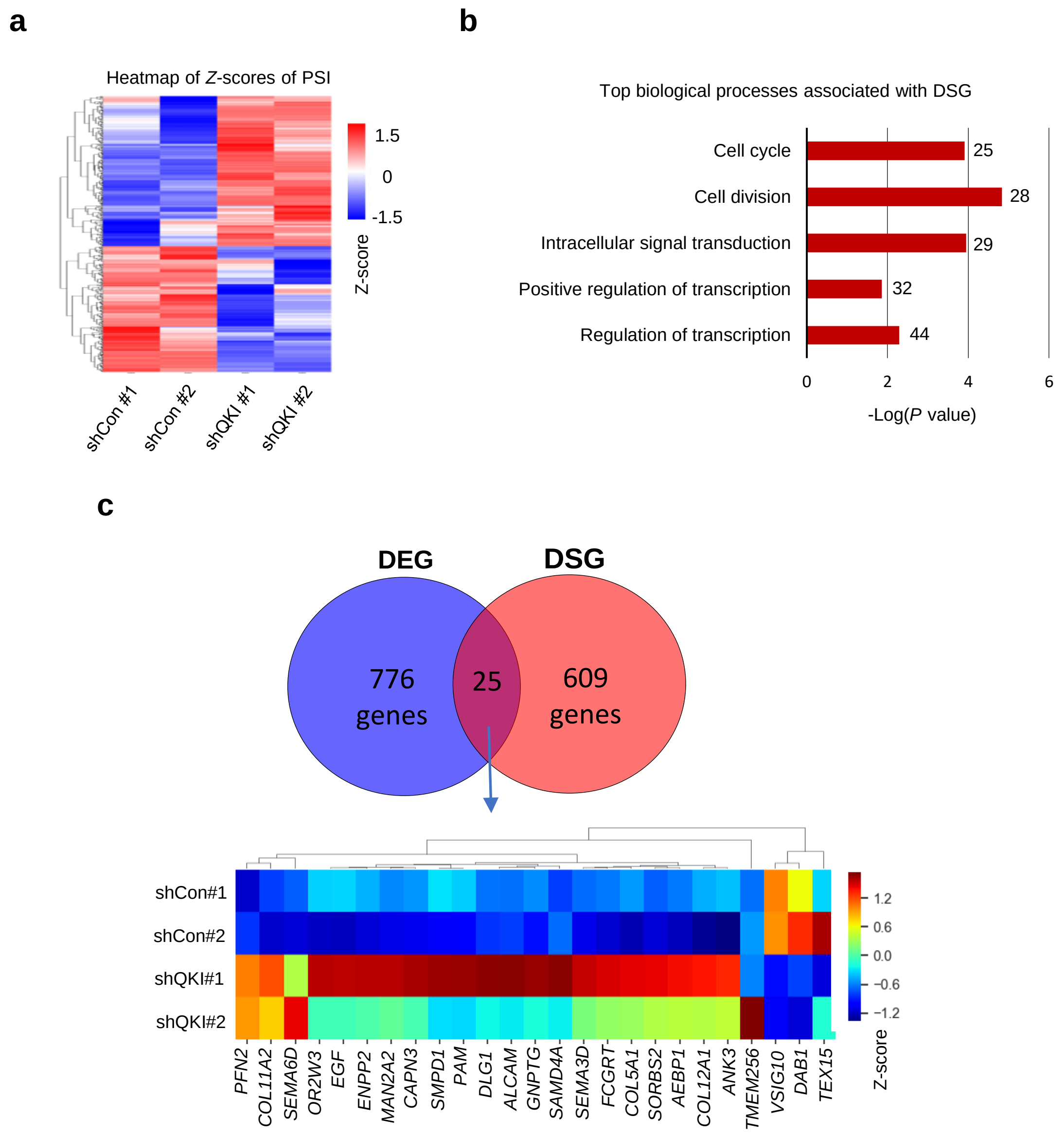

Supplementary Fig. 3 *QKI* regulates alternative splicing programs related to cell proliferation and glial lineage specification. a Heatmap of percent spliced-in (PSI) Z-scores for 634 differentially spliced exons (DSEs) in QKI-deficient SCPs (shQKI #1 and #2) relative to shCon controls (shCon #1 and #2). **b** GO enrichment analysis of genes with altered splicing, showing enrichment in biological processes such as cell cycle regulation, RNA metabolism, and cytoskeletal organization. **c** Venn diagram showing overlap between DEGs and DSEs, identifying 25 genes co-regulated at both transcriptional and post-transcriptional levels, many of which are implicated in proliferation and glial development.

Supplementary Fig. 4

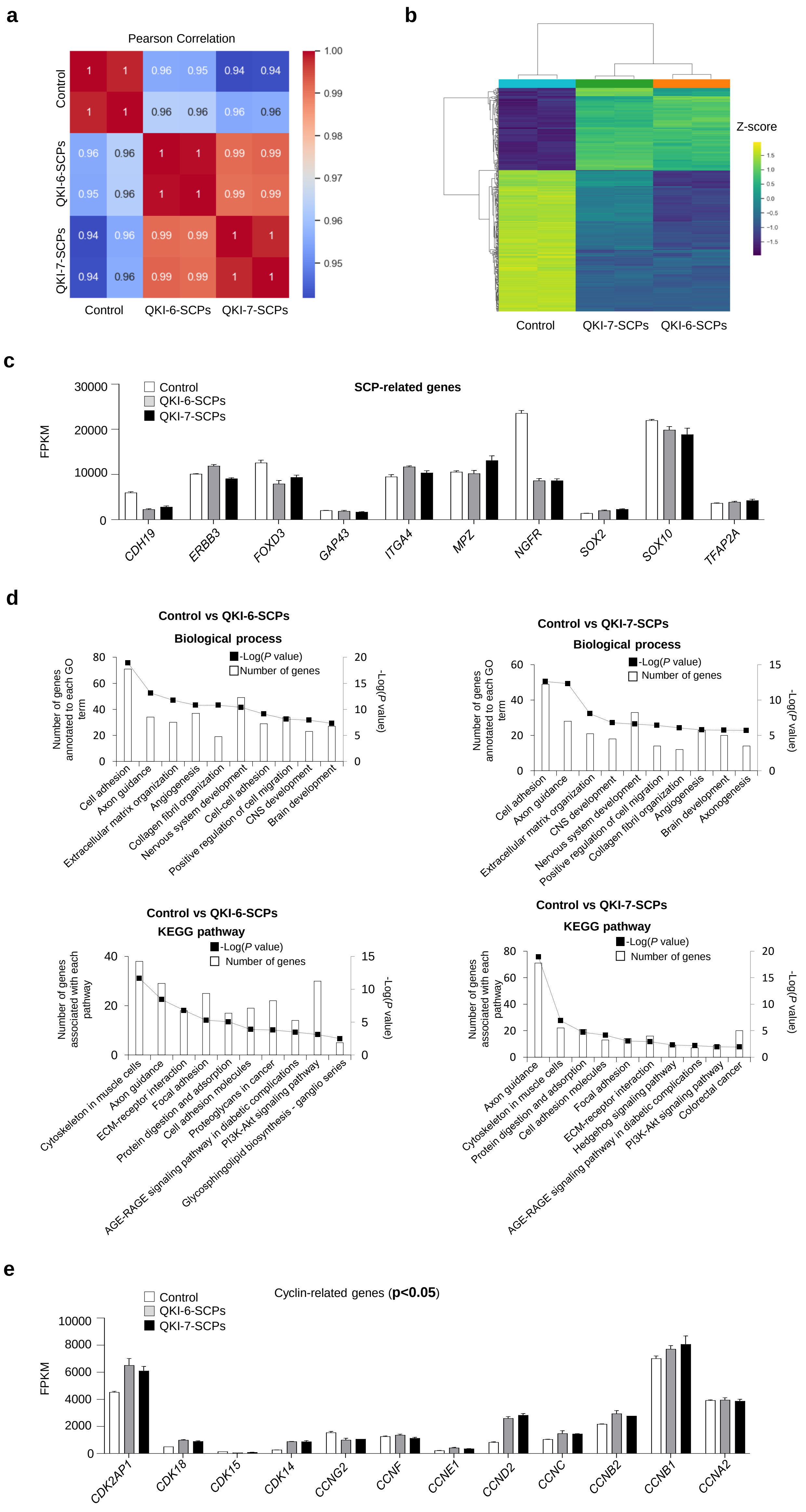

Supplementary Fig. 4 Comparative transcriptomic profiling of QKI-6- and QKI-7-overexpressing SCPs versus control SCPs. a Pearson correlation matrix comparing global transcriptomic profiles of control, *QKI-6*- (QKI-6-SCPs) , and *QKI-7*-overexpressing SCPs (QKI-7-SCPs). Correlation was calculated using genes with CPM > 10 after median normalization, revealing both shared and divergent transcriptomic signatures. **b** Hierarchical clustering heatmap of 808 DEGs (fold change > 2, $p < 0.05$), showing distinct transcriptional profiles in QKI-6- and QKI-7-SCPs compared to controls. **c** FPKM expression levels of canonical SCP marker genes across the three groups, confirming maintenance of SCP identity following *QKI* isoform overexpression. **d** GO enrichment analysis of upregulated and downregulated DEGs, highlighting the top 10 enriched biological processes. KEGG pathway analysis identifies major signaling and regulatory pathways modulated by *QKI-6* and *QKI-7*, with bar lengths indicating the number of DEGs per pathway. **e** FPKM values of representative cyclin genes involved in G1/S and G2/M transitions, demonstrating upregulation of proliferative regulators in both QKI-6- and QKI-7-SCPs, with a more pronounced effect observed in QKI-6-overexpressing cells.

Supplementary Fig. 5

a

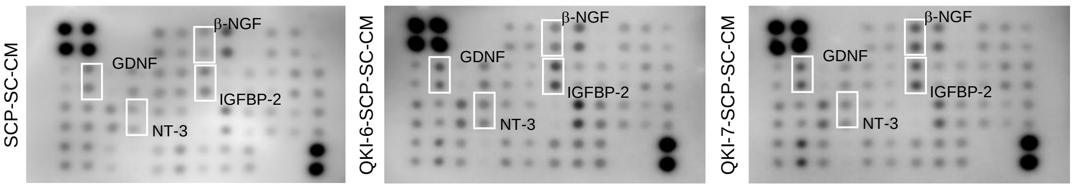

b

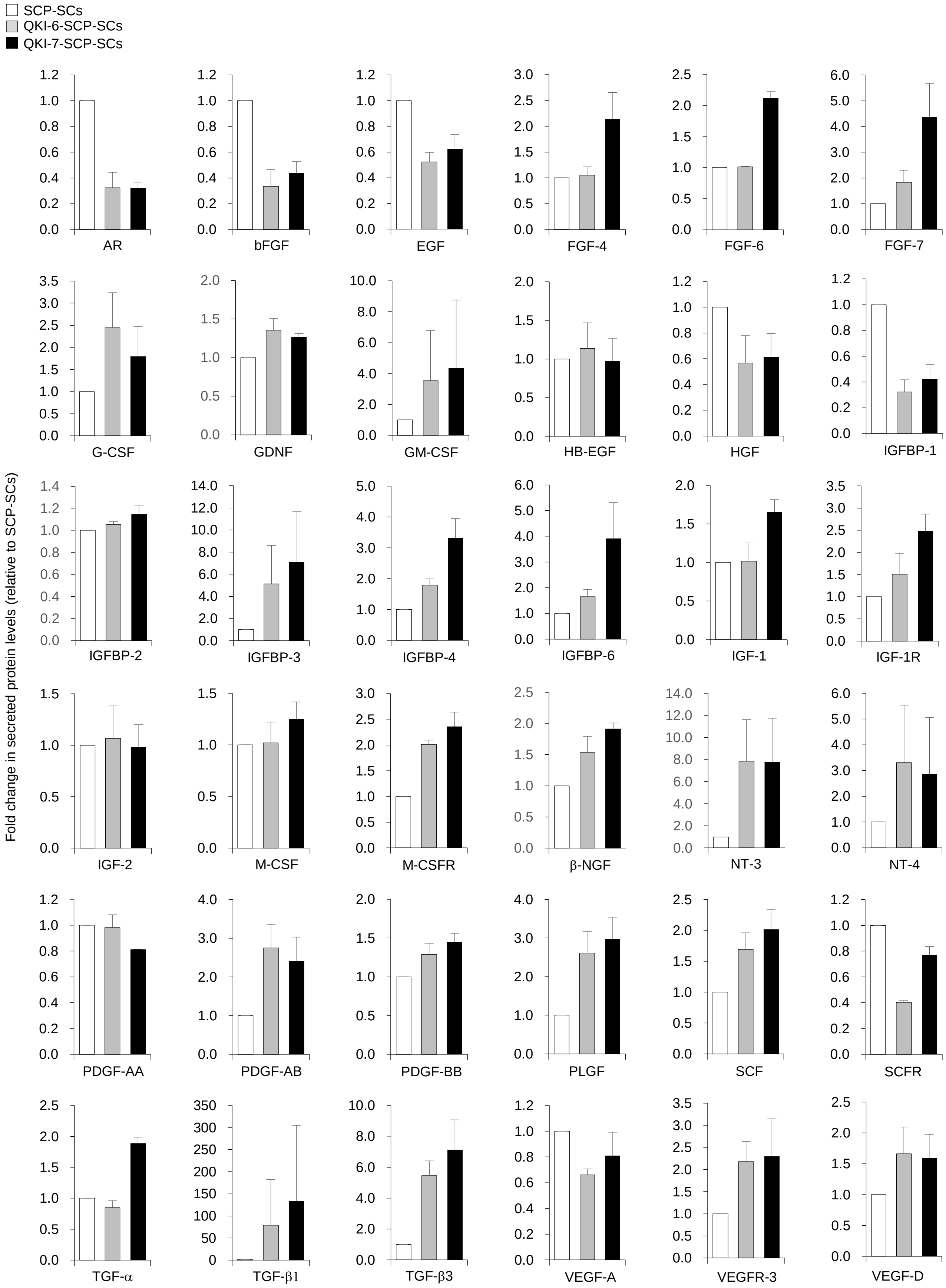

Supplementary Fig. 5 Growth factor profiling of conditioned media from QKI-overexpressing SCP-derived SCs. a Growth factor antibody array performed using conditioned media (CM) collected from control SCP-derived SCs (SCP-SC-CM), QKI-6-overexpressing (QKI-6-SCP-SC-CM), and QKI-7-overexpressing SCP-SCs (QKI-7-SCP-SC-CM). Each spot on the array represents a specific paracrine factor, with signal intensity reflecting its relative abundance. **b** Densitometric quantification was performed for 36 detected trophic factors, including AR, bFGF, EGF, FGF-4, FGF-6, -7, G-CSF, GDNF, GM-CSF, HB-EGF, HGF, IGFBP-1, -2, -3, -4, -6, IGF-I, IGF-IR, IGF-2, M-CSF, M-CSFR, β-NGF, NT-3, 4, PDGF-AA, PDGF-AB, PDGF-BB, PLGF, SCF, SCFR, TGF-α, TGF-β1, TGF-β3, VEGF-A, VEGFR-3, and VEGF-D (corresponding to Fig. 5L). Signal intensities were normalized to internal reference (REF) spots and quantified using ImageJ software. Data are presented as mean ± SD.

Supplementary Table 1. Primer sequences used for quantitative RT-PCR analysis

Gene	Sequence (5'-->3')	
BDNF	Forward	CTA CGA GAC CAA GTG CAA TCC
	Reverse	AAT CGC CAG CCA ATT CTC TTT
GAP43	Forward	GGC CGC AAC CAA AAT TCA GG
	Reverse	CGG CAG TAG TGG TGC CTT C
GAPDH	Forward	ACA ACT TTG GTA TCG TGG AAG G
	Reverse	GCC ATC ACG CCA CAG TTT C
GFAP	Forward	AGG TCC ATG TGG AGC TTG AC
	Reverse	GCC ATT GCC TCA TAC TGC GT
GDNF	Forward	GGC AGT GCT TCC TAG AAG AGA
	Reverse	AAG ACA CAA CCC CGG TTT TTG
IGFBP2	Forward	GAC AAT GGC GAT GAC CAC TCA
	Reverse	CAG CTC CTT CAT ACC CGA CTT
MBP	Forward	CAG CGT CAC GGC TCC AAA TA
	Reverse	CTG GCT ACG GGC ATG AGA C
MPZ	Forward	AAG TGC CAA CTA GGT ACG GG
	Reverse	CAT AGC ACT GAG CCT CCT CT
NGF	Forward	GGC AGA CCC GCA ACA TTA CT
	Reverse	CAC CAC CGA CCT CGA AGT C
NGFR	Forward	TGG CCT ACA TAG CCT TCA AGA
	Reverse	GAG ATG CCA CTG TCG CTG T
NT3	Forward	GAA CTG CTG CGA CAA CAG AGA
	Reverse	CCC ACG TAA TCC TCC ATG AGA
PLP1	Forward	ACC TAT GCC CTG ACC GTTG
	Reverse	TGC TGG GGA AGG CAA TAG ACT
PMP22	Forward	GAT CCT GTC GAT CAT CTT CAG C
	Reverse	AGC ACT CAT CAC GCA CAG AC
S100B	Forward	GAC CCT CAT CAA CGT GTT CCA
	Reverse	CCA CAA GCA CCA CAT ACT CCT
SOX10	Forward	CCT CAC AGA TCG CCT ACA CC
	Reverse	CAT ATA GGA GAA GGC CGA GTA GA
QKI-5	Forward	CTG TCA TGC CAA ACG GAA C
	Reverse	GAT GGA CAC GCA TAT CGT G
QKI-6	Forward	CTG TCA TGC CAA ACG GAA C
	Reverse	TTT CGT TGG GAA AGC CAT AC
QKI-7	Forward	CTG TCA TGC CAA ACG GAA C
	Reverse	CAT GAC TGG CAT TTC AAT CCA C

Supplementary Table 2. Primer sequences for alternative splicing validation

Gene	Sequence (5'-->3')	
ANK3	Forward	CAG TAG TGG ACA CCC TGA AGA
	Reverse	ACC TAT CCG AAC TGA AGG AGC
DLG1	Forward	AGG TCG GAG TGA TTC CCA GT
	Reverse	CGT ATT CTT CTT GAC CAC GGT
HAUS2	Forward	CGG GGA TGG TCA ATC AGG AG
	Reverse	TGC TAC AAA GTA ACT GCA AAA CA
NEK1	Forward	GAG TTC CTG CAG CGA AAA CG
	Reverse	TGC ATG GGC CTT CAG TGA TT
PLP1	Forward	GCC GGC TAC AAT TGG AGT CAG
	Reverse	AGG AGC CAC ACA ACG GTCA
PMP22 V1	Forward	AAG GGG TTA CGC TGT TTG G
	Reverse	GGA GGA CGA TGA TAC TCA GCA
PMP22 V2	Forward	ACC CGA GTT TGT GTT TGA GG
	Reverse	GGA GGA CGA TGA TAC TCA GCA
RAB11FIP3	Forward	TCC CCA GAG ACC CTA TGC AA
	Reverse	CAG CAA TGT CCT CCT CTG GG
STAG2	Forward	GGC TCC GCT TCT CTG TGT AG
	Reverse	CGG TTT GGA CCA GAA GGA GG

Supplementary Table 3. List of antibodies used for immunostaining and Western blotting

Protein	Company (Cat. No.)	Dilution
AKT	Cell signaling Technology (9272)	1:2000
BAX	Cell signaling Technology (12708)	1:1000
BCL2	Abcam (AB59347)	1:500
Cyclin B	Cell signaling Technology (4138)	1:1000
Cyclin E	SCBT (sc-247)	1:200
ERK	SCBT (sc-514302)	1:500
GAP43	Abcam (AB75810)	1:500
GAPDH	SCBT (sc-47724)	1:2000
Ki67	BD (550609)	1:50
NGF	Thermo Fisher Scientific (MA5-43760)	1:500
p27Kip1	Cell signaling Technology (3688)	1:1000
pAKT	Cell signaling Technology (9271)	1:1000
Pan-QKI	Abcam (AB126742)	1:1000
pERK	Cell signaling Technology (4370)	1:1000
QKI-5	Thermo Fisher Scientific (A300-183A)	1:2000
QKI-6	Neuromab (75-190)	1:500
QKI-7	Neuromab (73-200)	1:1000
S100B	Abcam (AB52642)	1:500
SOX10	Abcam (AB155279)	1:200
Goat Anti-Rabbit IgG H&L (Cy3 ®) preadsorbed	Abcam (AB6939)	1:400
Goat Anti-mouse IgG H&L (Cy3 ®) preadsorbed	Abcam (AB97035)	1:400
Alexa Fluor™ 488 donkey anti-mouse IgG (H+L)	Thermo Fisher Scientific (A21202)	1:400
Alexa Fluor™ 488 goat anti-rabbit IgG (H+L)	Thermo Fisher Scientific (A11008)	1:400
Goat anti-mouse IgG HRP	Thermo Fisher Scientific (31430)	1:5000
Goat anti-rabbit IgG HRP	Thermo Fisher Scientific (32260)	1:5000
